# Simultaneous detection and differentiation of *Mycobacterium tuberculosis* and nontuberculous mycobacteria in smear-negative sputum by a multiplex PCR assay: a clinical feasibility study

**DOI:** 10.1128/spectrum.02316-24

**Published:** 2025-05-30

**Authors:** Long Xie, Jing Cao, Yong Yang, Shao-Long Jiang, Jin-Lin Tan, Jia Zhang, Ze-Fan Ruan, Li Chen, Da-Yong Xu, Zhong Chen, Ming-Xiang Huang, Xi-Wen Jiang

**Affiliations:** 1 Translational and Clinical Research Institute, Faculty of Medical Sciences, Newcastle University 151515 , Newcastle upon Tyne, United Kingdom; 2 The First Hospital of Changsha and Affiliated Changsha Hospital, Xiangya Medical School, Central South University 12570, Changsha, China; 3 Research Institute, DAAN Gene Co., Ltd. 637703 , Guangzhou, China; 4 The Medicine and Biological Engineering Technology Research Centre of the Ministry of Health, Guangzhou, China; 5 Chaoshan Hospital, The First Affiliated Hospital of Jinan University, Chaozhou, China; 6 The First Affiliated Hospital of Jinan University, Guangzhou, China; 7 Fuzhou Pulmonary Hospital & Fujian Medical University Clinical Teaching Hospital, Fujian Medical University 74551, Fuzhou, China; 8 School of Life Sciences and Biopharmaceutics, Guangdong Pharmaceutical University 71237, Guangzhou, China; City of Hope Department of Pathology, Duarte, California, USA

**Keywords:** *Mycobacterium tuberculosis*, nontuberculous mycobacteria, multiplex TaqMan PCR, assay development and clinical feasibility evaluation, sputum detection

## Abstract

Accurate and rapid differentiation of *Mycobacterium tuberculosis* (*M. tuberculosis*) and nontuberculous mycobacteria (NTM) remains challenging, especially in smear-negative samples. Routine diagnostic methods often lack specificity and rapidity, highlighting the urgency of developing applicative molecular diagnostics to guide clinical management and improve patient outcomes. We developed and validated a multiplex PCR assay employing TaqMan probes targeting the *IS6110* and *rpoB* genes to simultaneously detect *M. tuberculosis* and 23 NTM species in a single reaction, while avoiding cross-reactivity. Analytical performance was assessed using reference strains, and clinical feasibility was evaluated in a prospective, single-center pilot study involving 96 smear-negative presumptive tuberculosis patients. The assay’s diagnostic performance was compared to mycobacterial culture, DNA sequencing, and the Xpert MTB/RIF assay. The multiplex PCR assay demonstrated a wide linear dynamic range (10^8^–10^3^ copies/mL), low limit of detection (10^3^ copies/mL), high precision (intra-assay CV 0.05–1.18%, inter-assay CV 0.08–2.57%), and complete specificity for both *M. tuberculosis* and NTM. In the clinical feasibility study, using a combination of clinical diagnosis and mycobacterial culture as reference standards, it achieved sensitivities of 73.33% (*M. tuberculosis*) and 100% (NTM), and specificities of 100% (*M. tuberculosis*) and 94.33% (NTM). Notably, it reliably identified *M. tuberculosis*/NTM co-infections. The Multiplex PCR assay fills a critical gap in the rapid, accurate diagnosis of *M. tuberculosis* and NTM in challenging clinical specimens. With a cost of $5 per test and a turnaround time of 3 h, our assay has the potential to improve patient care and optimize mycobacterial infection treatment strategies in resource-limited settings.

**IMPORTANCE:**

Rapid and accurate differentiation between *Mycobacterium tuberculosis* (*M. tuberculosis*) and nontuberculous mycobacteria (NTM) is essential for ensuring timely and appropriate treatment, especially in high tuberculosis (TB) burden regions. Conventional diagnostic methods, such as smear microscopy and culture, often lack the sensitivity or speed needed for reliable results in smear-negative cases, risking misdiagnosis and delayed care. In this study, we developed a novel multiplex real-time PCR assay capable of simultaneously detecting *M. tuberculosis* and up to 23 clinically relevant NTM species with high specificity and sensitivity. By targeting distinct genetic markers for *M. tuberculosis* and NTM, our assay provides a cost-effective, 3-h diagnostic solution that enhances diagnostic accuracy in challenging samples. This innovation addresses a critical gap in mycobacterial diagnostics, supporting improved patient outcomes and aligning with global health priorities for the control and elimination of TB.

## INTRODUCTION

Tuberculosis (TB), caused by the *Mycobacterium tuberculosis* (*M. tuberculosis*) complex (MTBC), remains a formidable global health challenge (1). The World Health Organization (WHO)’s 2024 Global Tuberculosis Report 2024 estimates 1.25 million deaths and 10.8 million new infections in 2023 (2), which represents an increase from 10.7 million in 2022, indicating ongoing community transmission risks. In 2023, China was the third highest TB-burdened country, reporting approximately 741,000 new cases (6.8% of the global total) and an incidence rate of 52 per 100,000 (2). These statistics highlight the ongoing difficulties in global TB control.

Concurrently, clinical concerns regarding nontuberculous mycobacteria (NTM) are escalating. These organisms, distinct from MTBC (3, 4), are increasingly isolated from patients, indicating a rising prevalence of NTM disease under predisposing conditions (5). While environmental resources are presumed, transmission modes require further investigation (6). Common NTM species associated with pulmonary diseases include *Mycobacterium avium*, *Mycobacterium abscessus*, *Mycobacterium kansasii*, and *Mycobacterium intracellulare* (7–9). The incidence of pulmonary NTM infections is rising globally, including in China (8), where significant co-infection rates (approximately 3%) with *M. tuberculosis* have been observed in patients initially suspected of multidrug-resistant TB (10).

NTM infections present clinical manifestations similar to TB, particularly in individuals with comorbidities like chronic obstructive pulmonary disease (COPD), pneumoconiosis, bronchiectasis, or HIV, who are vulnerable to both mycobacterial types (11). A primary challenge is the deficiency of accurate, sensitive methods to differentiate *M. tuberculosis* from NTMs, leading to misdiagnosis and undetected co-infections (12). Morphological similarities further complicate differentiation in clinical tests (13). In many low- and middle-income countries, including China, acid-fast bacilli (AFB) smear microscopy is common for TB diagnosis (14) but has low sensitivity (20–60%) and necessitates a high bacillary load (≥10,000 bacilli/mL sputum) for positivity, resulting in missed diagnoses (14). While selective media containing *p*-nitro benzoic acid (PNB) can facilitate differentiation by promoting NTM growth while inhibiting *M. tuberculosis*, conventional mycobacterial culture is time-consuming and labor-intensive (13).

The resistance of most NTM to anti-TB drugs necessitates distinct management strategies, especially in co-infected patients, complicating treatment (15, 16). Delays in accurate bacterial identification hinder effective treatment and exacerbate disease progression. The American Thoracic Society and the Infectious Diseases Society of America (ATS/IDSA) guidelines emphasize the necessity for precise diagnostics, promoting personalized NTM therapeutic regimens based on drug susceptibility and clinical context (13, 17). The 2020 revision highlights the importance of accurate diagnostics in managing NTM infections (13). There is an unmet need for clinical laboratory methods capable of rapid and accurate differentiation between *M. tuberculosis* and NTM infections. In response, the WHO endorses innovative molecular assays as alternatives to smear microscopy, particularly in peripheral settings (18), detailing target product profiles (TPPs) that outline desirable performance characteristics: high specificity (>98%), and sensitivity surpassing sputum smear microscopy (>60%), especially when sputum smears are negative. Robustness in challenging environmental conditions, simplicity for minimally trained personnel, and affordability (<$6.00 per test) are essential for widespread implementation (19, 20).

Nucleic acid amplification test, particularly fluorescent quantitative polymerase chain reaction (PCR), has gained traction for mycobacterial detection, offering improved sensitivity and specificity, and reduced cross-contamination risk by eliminating post-amplification processing (18, 21–24). Several laboratory-developed multiplex real-time PCR assays have been reported for differentiating *M. tuberculosis* from NTM. However, many target a limited range of common NTM species or employ complex amplification protocols, potentially complicating diagnosis and prolonging turnaround times (21, 22, 24, 25). Validation has predominantly been restricted to cultured isolates or pre-selected positive clinical specimens (22, 25), potentially overestimating performance compared to real-world diagnostic scenarios involving direct testing of specimens like smear-negative sputum. Commercially available automated platforms, such as Roche Diagnostics’ cobas MTB, Hain Lifescience/Bruker’s FluoroType MTBDR, and Cepheid’s GeneXpert MTB/RIF, offer integrated solutions (26–29) but primarily target *M. tuberculosis*, lacking comprehensive NTM identification capabilities critical for detecting mixed infections. Their closed-system nature can also limit adaptability in routine laboratory settings.

To assess these limitations, we developed and validated a multiplex real-time PCR assay for accurately differentiating *M. tuberculosis* from recognized NTM species. Our assay, termed Multiplex PCR MTB/NTM, targets the insertion sequence 6110 (*IS6110*) gene, a transposable genetic sequence specific to MTBC (30), for sensitive *M. tuberculosis* detection and conserved yet variable regions within the *rpoB* gene (encoding the RNA polymerase β-subunit) (31) for the specific identification of 23 NTM species (listed in Table S1), while ensuring discrimination from *M. tuberculosis* sequences. Compatible with widely available and cost-effective real-time PCR instruments. Our assay is particularly beneficial for peripheral health centers that often lack advanced diagnostic facilities like bronchoscopy. It facilitates rapid and precise identification of 23 NTM species and *M. tuberculosis*, adhering to WHO’s TPP guidelines with a projected manufacturing cost under $5.00 per test. Following successful preliminary validation in detecting *M. tuberculosis* using residual nucleic acids in sputum from confirmed TB patients (32), we conducted a pilot clinical feasibility evaluation. This crucial step involved applying our assay to AFB smear-negative sputum samples from suspected first-time TB patients, a challenging scenario characterized by low bacterial loads. Assay performance was evaluated by comparing outcomes against DNA sequencing, mycobacterial culture, and Cepheid’s Xpert MTB/RIF, using the final clinical diagnosis as the reference standard to assess its real-world diagnostic sensitivity and specificity.

## MATERIALS AND METHODS

### Participants and study design

**Fig 1:**
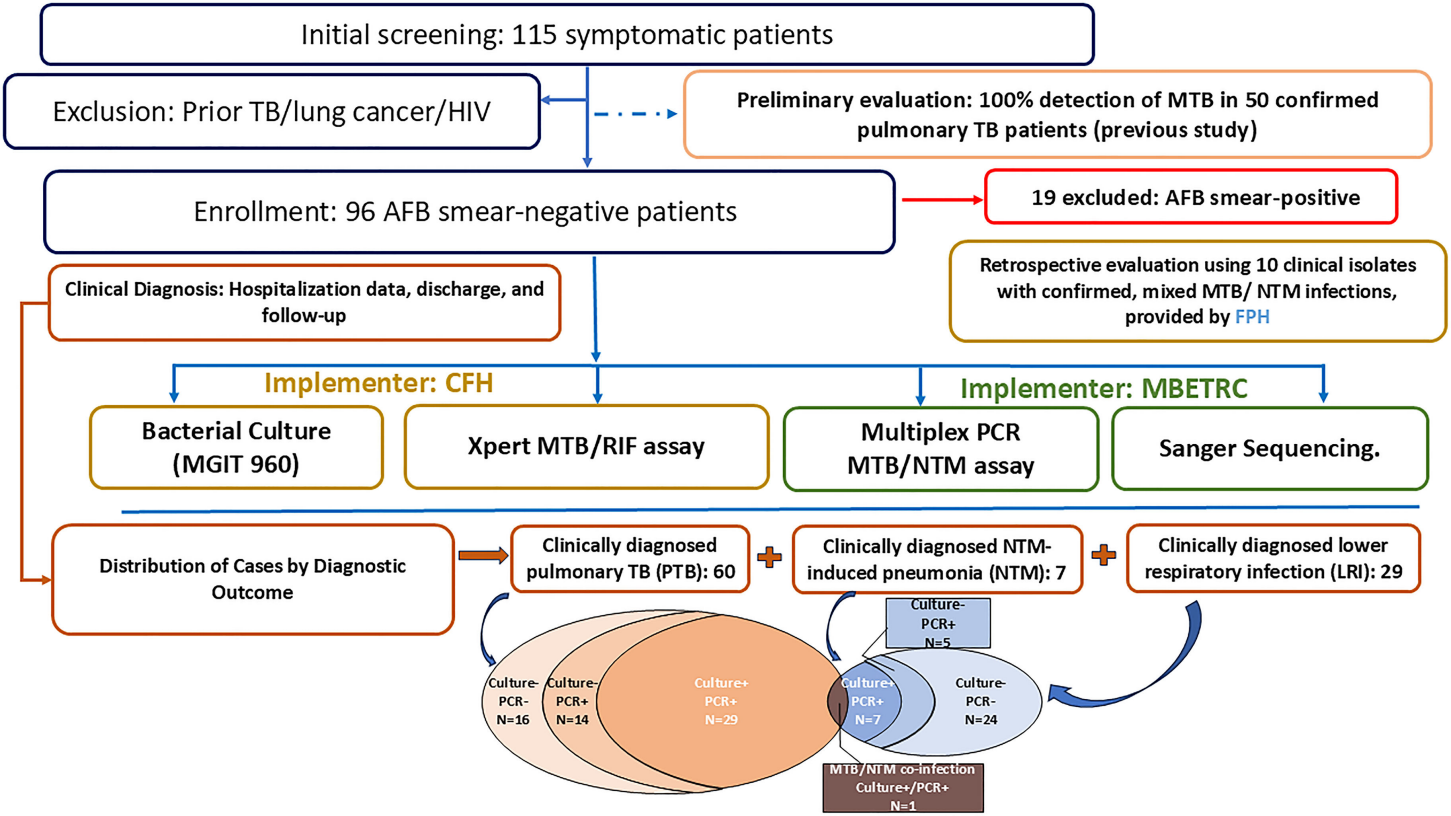
The pilot clinical feasibility evaluation study design and participant flow. This flowchart illustrates participant recruitment, preliminary/retrospective assay validation, inclusion/exclusion criteria, the various testing procedures and their implementers, and the final diagnostic classification of the 96 enrolled participants. Nineteen patients were excluded due to positive AFB smears, resulting in the enrollment of 96 AFB smear-negative participants. Sputum samples from enrolled patients underwent parallel testing: Mycobacterial Culture (MGIT 960) and Xpert MTB/RIF assay performed at the First Hospital of Changsha (CFH); Multiplex PCR MTB/NTM assay and Sanger Sequencing performed at the Medicine and Biological Engineering Technology Research Centre (MBETRC). Final clinical diagnosis, based on hospitalization data, discharge diagnosis, response to therapy, and 3-month follow-up incorporating culture results according to established guidelines (13, 33, 34), served as the reference standard. This classified participants into: 60 clinically diagnosed pulmonary TB (PTB) cases (including one *M. tuberculosis*/NTM co-infection identified by PCR and culture), 7 NTM-induced pneumonia cases (confirmed by culture), and 29 lower respiratory tract infection (LRTI) cases due to other pathogens. Venn diagrams illustrate the distribution of Multiplex PCR MTB/NTM assay results relative to culture outcomes within the PTB and NTM groups.

This two-phase study, led by the Medicine and Biological Engineering Technology Research Centre of the Ministry of Health (MBETRC), involved assay development followed by a prospective pilot clinical feasibility evaluation conducted at the First Hospital of Changsha (CFH) between 1 February and 31 October 2023. The overall study design, patient recruitment flow, testing procedures, and diagnostic outcomes are schematically represented in Fig. 1. Briefly, after initial assay validation using reference strains (Table S1) and a preliminary evaluation with DNA samples from 50 TB patients with AFB smear positivity (32), we prospectively screened 115 hospitalized patients exhibiting symptoms suggestive of pulmonary TB (e.g., fever, cough, and sputum production). Patients with prior TB, lung cancer, or HIV infection were excluded. Following informed consent, each patient provided a single sputum specimen (≥5 mL) for smear microscopy screening. After excluding 19 patients with positive results, 96 smear-negative individuals were enrolled without demographic restrictions. The remaining initial samples were used for all analyses, including the Multiplex PCR MTB/NTM assay, mycobacterial culture, Sanger sequencing, and Xpert MTB/RIF. Participants also provided a second sputum sample one week post-enrollment for repeat smear and culture. Comprehensive medical evaluations, including high-resolution computed tomography (CT), assessed clinical status.

Pulmonary TB diagnostic criteria followed expert consensus from China’s national “Clinical Practice Guidelines for Early Detection of Pulmonary Tuberculosis (33)” and the WHO’s operational handbook on TB (34). The final clinical TB diagnosis incorporates clinical presentation, radiological evidence, microbiology, response to specific therapy, and 3-month telephone and in-person interview follow-up. For definitive NTM pulmonary infection diagnosis, we adhered to the ATS/IDSA’s 2020 criteria (13), which mandates bacterial culture detection of NTM species. These diagnoses served as the reference standard for evaluating diagnostic performance.

### Sample size calculation

This prospective pilot clinical feasibility study’s sample size can be flexible, ranging from 10 to 300 participants, according to established criteria (35). The area under the curve (AUC) of the receiver operating characteristic (ROC) curve served as the primary indicator of diagnostic accuracy. An enhanced formula from a previous study (36) was utilized for sample size calculation based on AUC, targeting an AUC of 0.85 with a 0.1 error margin, yielding 84 participants (including cases and controls) after adjusting for a 10% dropout rate (details in Supplementary information additional file 1)

### Sputum processing, smear microscopy, and mycobacterial culture

Sputum samples (approximately 5.0 mL each) were processed in the CFH’s. Biosafety Level-3 (BSL-3) Laboratory per Chinese TB Laboratories Guidelines, adapted from WHO recommendation (37). Samples were digested and decontaminated using a modified Petroff’s method: 5 mL sputum mixed with 10 mL of 0.5% N-acetyl-l-cysteine/3% sodium hydroxide (NaLC-NaOH) solution for 15 min, then diluted to 45 mL with phosphate-buffered saline (PBS) and centrifuged at 3,000 × *g* for 15 min. The pellet was resuspended in 2.7 mL PBS, and 0.2 mL of each suspension was examined via direct smear microscopy using the standard Ziehl Neelsen’s staining method, as previously reported (38). Only smear-negative samples (no AFB observed in 300 fields at 1,000× magnification) proceeded to enrollment for further processing. A 0.5 mL aliquot was inoculated into the BACTEC MGIT 960 system (Becton Dickinson, Sparks, MD) for up to 6 weeks or until flagged positive. Positive cultures were first examined by AFB smear microscopy, and *M. tuberculosis* was verified using an immunochromatographic method with the MPT64 antigen detection kit by Bioline TBAgMPT64 (Standard Diagnostics, Seoul, South Korea). Differentiation between *M. tuberculosis* and NTM was performed by inoculating samples into growth media containing 0.5 mg/mL PNB. Additionally, 1.0 mL of each suspension was tested with the Xpert MTB/RIF assay. Processed sputum sediments (0.5 mL) were transported to the MBETRC and kept at 4°C. All specimens arrived within 48 h of collection and were processed within 12 h of arrival. Each 0.5 mL of sediment was divided into five tubes of 100 µL each, with two aliquots undergoing DNA extraction. The remaining portions were stored at −80°C for further testing. To minimize bias, personnel handling bacterial culture at the CFH were blinded to molecular testing results, and molecular testing staff were kept uninformed about culture outcomes.

### Bacterial strain and clinical isolates

During the analytical validation of our multiplex PCR assay, we utilized 39 reference strains, 10 clinical isolates representing *M. tuberculosis*/NTM co-infections, and human HeLa cells. The reference strains comprised 24 mycobacterial strains and 15 bacterial strains as negative controls for cross-reactivity assessment (Table S1). These reference strains were purchased from various institutions, including the American Type Culture Collection (ATCC; Manassas, VA), the National Institute for Food and Drug Control (https://www.nifdc.org.cn/nifdc/, Beijing, China), the Culture Collection University of Gothenburg (Göteborg, Sweden), the Guangdong Microbial Culture Collection Centre (Guangzhou, China), and the National Centre for Medical Culture Collections (CMCC; Beijing, China). Due to the absence of BSL-3 facilities at the MBETRC, the *M. tuberculosis* standard strain selected was the virulence-attenuated H37Ra (ATCC25177) instead of the virulent H37Rv strain. The clinical isolates, identified by Fuzhou Pulmonary Hospital (FPH), comprised various co-infections: 5 *M*. *tuberculosis*/*M. intracellulare*, 2 *M*. *tuberculosis*/*M. avium*, 1 *M*. *tuberculosis*/*M. kansasii*, 1 *M*. *tuberculosis*/*M. abscessus*, and 1 *M*. *tuberculosis*/*Mycobacterium malmoense* (10). Following clinical testing, our assay kit was delivered to FPH for validation of its diagnostic capacity on co-infected samples by molecular laboratory professionals conducting retrospective experiments. Mycobacterial strains were lyophilized and stored at −80°C, with recovery achieved via culturing on Middlebrook 7H10 agar medium at 37°C. Clinical isolates were initially preserved at −80°C in Middlebrook 7H9 broth with 10% glycerol and later recovered on Löwenstein-Jensen (L-J) medium for 4 weeks at 37°C. To monitor nucleic acids extraction efficiency from human samples and PCR amplification, a human HeLa cell line (ATCC reference number CCL-2) stably expressing RHOG, a member of the Rho family of small GTPases, was cultured following supplier protocol, with the supernatant harvested, clarified, diluted to approximately 1 × 10^8^ cells/mL, and stored at −80°C.

### DNA extraction

A systematic extraction protocol was implemented across reference strains, clinical isolates, sputum sediments, and cultured cells. The automated Smart32 Nucleic Acid Extraction instrument and its Nucleic Acid Isolation Kit (Da’an Gene, Guangzhou, China) were utilized following the manufacturer’s manuals. Bacterial samples were prepared by resuspending colonies from freshly cultured bacteria in 500 µL Tris-EDTA (TE) buffer and heating at 100°C for 30 min, followed by centrifugation at 13,000 rpm for 5 min. Supernatants containing genomic DNA were stored at −20°C. The initial volume for DNA extraction was standardized at 200 µL for various biological substances, except for sputum samples, where sediment suspension was used directly. The extraction process took approximately 40 min per sample batch, with DNA eluted in 60 µL and stored at −20°C until further analysis.

### Design of primer-probe pairs for the multiplex PCR MTB/NTM assay

**Fig 2:**
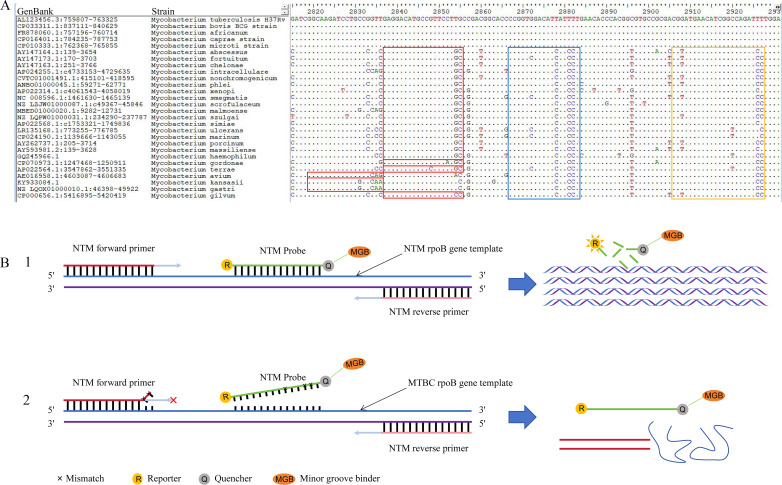
Primer and probe design targeting the *rpoB* gene for specific identification of NTM species. (**A**) Sequence alignment and primer/probe design. This panel displays a detailed sequence alignment of the *rpoB* gene regions across various *Mycobacterium* strains used for designing the primers and probe. Sequences within the red box were chosen for the forward primer, designed to ensure specificity to NTM species by incorporating two to three consecutive mismatches at the 3′ end when binding to the *M. tuberculosis rpoB* gene template, thereby effectively preventing amplification. These forward primers for the NTM *rpoB* gene encompass six sequences compatible with 23 NTM species. Sequences within the blue box were selected for the probe, which includes three base mismatches when hybridized to the *M. tuberculosis rpoB* gene template. A minor groove binder (MGB) was incorporated to lower the probe’s melting temperature (*T*_m_) below the PCR annealing/extension temperature, preventing non-specific binding and hydrolysis with the *M. tuberculosis* template and subsequent cleavage of the probe, thus avoiding fluorescence signal generation. (**B**) Schematic representation of the TaqMan probe-based PCR assay. This schematic illustrates two scenarios: (i) Specific amplification: when the NTM probe and primers hybridize to the NTM *rpoB* gene template, normal amplification, and fluorescence signal generation occur. (ii) Non-specific binding prevention: when the NTM probe and primers encounter the *M. tuberculosis rpoB* gene template, the mismatches prevent proper binding and hydrolysis, resulting in no fluorescence signal and no amplification of the target.

TaqMan probes were designed for simultaneous identification of target genes in *M. tuberculosis* and NTM, along with the endogenous reference gene RHOG. The *IS6110* and *rpoB* genes were selected as individual targets for *M. tuberculosis* and NTM, respectively, simplifying the multi-target detection system and utilizing multi-color fluorescence channels of mainstream PCR instruments for signal amplification. Primer-probe pairs were designed to exploit unique genetic signatures, ensuring specific amplification regions within the *M. tuberculosis* and NTM genomes to avoid non-specific amplification. Notably, the *IS6110* primers and probes targeted the complete genomic sequence of the H37Rv strain to enhance diagnostic efficacy in TB patients, subsequently validated with DNA from the attenuated H37Ra strain. For *rpoB*, six forward primers targeted conserved sequences flanking variable regions across 23 NTM species, incorporating 3′ mismatches to the *M. tuberculosis* template. A TaqMan minor groove binder (MGB) probe with three intentional mismatches to the *M. tuberculosis* template, complemented by a universal reverse primer, was designed for stringent specificity of NTM *rpoB* while preventing *M. tuberculosis* amplification. Figure 2 schematically illustrates the design concept of the *rpoB* primer/probe by incorporating mismatches on the genomic DNA template. The primer and probe sequences (Table S2) were derived from the International Nucleotide Sequence Database Collaboration at the National Centre for Biotechnology Information (NCBI), analyzed utilising Oligo7 (http://oligo.net). Validation tests included specificity assessments via the Basic Local Alignment Search Tool (BLAST; http://blast.ncbi.nlm.gov/Blast.cgi), evaluation of internal primers hairpin, primer-dimer potential, G-C content and melting temperatures of the primers and probes using Oligo7, and sequence comparison analysis using Bioedit (https://bioedit.software.informer.com/). BLAST confirmed the conservation of the amplified *IS6110* region between H37Rv and H37Ra strains, validating the appropriateness of H37Ra for assay development (data not shown). TaqMan probes were individually labelled with diverse reporting dyes, including FAM, Texas Red, and CY5 on the 5′ end and quencher dye Black Hole Quencher 1 or 2 (BHQ-1 and BHQ-2) on the 3′ end. All primers and probes were synthesized by Da’an Gene (Guangzhou, China).

### Establishment of the multiplex real-time PCR

The assay development comprised the following three phases.

#### Primer/probe screening and validation

Candidate sets targeting *IS6110*, *rpoB,* and RHOG genes were screened via singleplex real-time PCR to optimize conditions (primer/probe annealing time/temperature, primer and probe concentrations, suitable buffer, and magnesium concentration). Specificity in *rpoB* gene primer/probe selection was critical for differentiating *M. tuberculosis* from NTM. A commercially engineered plasmid containing the *rpoB* sequence from *M. tuberculosis* H37Rv (NCBI GenBank accession number: AL123456.3), spanning 1,020 base pairs and homologous to the amplified regions of NTM species, was constructed from Sangon Biotech (Shanghai, China) using standard protocols. Amplified DNA sequences were inserted into the pUC57-kan vector by homologous recombination and enzymatic ligation, followed by transformation into *Escherichia coli* for cloning, with plasmid integrity verified by Sanger’s sequencing. Droplet digital (dd)PCR precisely quantified plasmid concentration, evaluating candidate primer/probe sets at 10^9^, 10^10^, and 10^11^ copies/mL. Sets that did not amplify the *rpoB* gene from the *M. tuberculosis* plasmid were deemed highly specific against 23 NTM species.

#### Multiplex optimization

The best-performing primer/probe sets were advanced to multiplex PCR screening under uniform reaction conditions, with the top three incorporated into a single-tube multiplex PCR assay.

#### Multiplex reaction and performance evaluation

The finalized multiplex real-time PCR was conducted in a 25 µL mixture containing 3 U Hot-Start Taq DNA polymerase, 0.5 U Uracil-DNA-Glycosylase (UDG) (all supplied by Da’an Gene, China), 65 mM Tris-HCl (pH 8.8), 2 mM MgCl_2_, 5% (vol/vol) Tween-20, 240 nM dNTPs (all purchased from Thermo-Fisher Scientific, Waltham, MA), primer concentrations ranging from 150 to 300 nM, 100 nM TaqMan probes, 5 µL template DNA, and diethyl pyrocarbonate-treated water. Reactions were performed on an ABI 7500 system (Thermo-Fisher Scientific, Waltham, MA) with cycling conditions: 2 min at 50°C for UDG reaction to prevent carry-over contamination, 15 min at 95°C for denaturation, followed by 40 cycles of 15 s at 95°C and 45 s at 55°C for annealing/extension. Fluorescence was recorded during annealing. Linearity and amplification efficiency were evaluated using gradient samples from reference strains (listed in Table S1) quantified by ddPCR, generating standard curves from serial 10-fold dilutions of template DNA at concentrations ranging from 10^8^ to 10^2^ copies/mL. Each multiplex PCR run included negative (double distilled deionized water) and positive controls (nucleic acids from reference strains at known concentrations). All samples were tested in triplicate unless otherwise stated. The entire experimental process, from nucleic acid extraction to signal generation, took approximately 3.0 h.

#### Result interpretation

Detection of target genes utilized standard fluorescence channels: FAM (*IS6110*), Texas-Red (*rpoB*), and CY5 (RHOG). The instrument’s integrated software automatically adjusted the cycle threshold (Ct) value to align with the exponential phase of amplification curves. A Ct value < 38 indicates positivity. *M. tuberculosis* detection was confirmed with amplification in FAM and CY5 channels below the defined cut-off. NTM presence was indicated by signals in Texas-Red and CY5 channels. Concurrent detection of both targets occurred when all three channels exhibited amplification signals below the threshold. A valid negative result was ascertained when only the CY5 channel showed an amplification curve with a valid Ct value. Amplification in FAM and/or Texas-Red channels without concurrent CY5 amplification rendered the test invalid, necessitating repetition for accuracy.

### Absolute quantification

ddPCR was utilized for precise quantification of DNA copy numbers from reference strains, as previously described (39). Procedures are detailed in Supplementary information additional file 1, with primer and probe sequences listed in Table S3.

### Evaluation of analytical performance

#### Linearity range and limit of detection (LOD)

Analytical performance was evaluated for the Multiplex PCR MTB/NTM assay, utilizing ddPCR for quantifying nucleic acid from *M. tuberculosis* and 23 NTM strains as controls. Ten-fold dilution standard curves were constructed for each strain, spanning 10^8^–10^2^ copies/mL, including single-target samples, dual-target mixtures of NTM strains spiked into *M. tuberculosis*, and a composite sample containing all 24 pathogens. Serial dilutions were assessed in single-plex and multiplex PCR to determine the assay’s linear range, with Ct values plotted against logarithmic dilution factors to generate standard curves. Efficiency (E) was calculated using the formula: *E* = 10^(−1/slope)^ – 1. A preliminary LOD prediction interval of 250–2,000 copies/mL was established for *M. tuberculosis* and NTM targets, refined through gradient dilution testing. The candidate LOD for *M. tuberculosis* was the lowest concentration consistently detected, while the minimal detectable concentration for NTM targets was determined across all species. These LODs were identified through three repetitive experiments and subsequently validated in 20 repeated tests, achieving a 95% detection success rate (19 out of 20 positive detections).

#### Reproducibility and precision

Assay precision was evaluated by measuring intra-assay and inter-assay coefficient of variation (CV). Three concentrations (10^7^, 10^5^, and 10^3^ copies/mL) were tested in triplicate for intra-assay reproducibility. Inter-assay variability was assessed by repeating the intra-assay experiment over three consecutive days. Means, standard deviation (SD), and CV of Ct values were calculated for each replicate set.

#### Specificity

Specificity was evaluated by simultaneously detecting 15 common gram-positive and gram-negative bacteria (Table S1) to exclude cross-reactivity, testing a mixture of nucleic acids at 10^5^ copies/mL alongside target DNA templates, with RHOG-specific primer/probe amplifying the endogenous internal control in human DNA from HeLa cells to prevent false-negative reactions.

### Diagnostic performance evaluation

A pilot clinical feasibility study assessed the diagnostic capability of the developed Multiplex PCR MTB/NTM assay, following previously detailed patient and study design protocols. Sputum aliquots from 96 AFB smear-negative cases were analyzed. Post-DNA extraction, samples were processed to detect *M. tuberculosis* and NTM via the PCR assay. A subset of DNA samples underwent concurrent sequencing for validation. Sputum aliquots were cultured at the CFH, furnishing a clinical diagnostic benchmark for the multiplex PCR assay’s performance. Efficacy was compared to the Xpert MTB/RIF assay, using separate sputum aliquots at the CFH, demonstrating that our assay provides diagnostic accuracy for *M. tuberculosis* detection comparable to existing molecular tools.

### DNA sequencing

Targeted *IS6110* and *rpoB* gene sequences were sequenced as previously reported (32). PCR primer sequences for sequencing are listed in Table S3. The BLAST algorithm confirmed *M. tuberculosis* or NTM identity in clinical samples through multiple alignments in the NCBI database. Closest matching species and their Genbank accession numbers, along with representative sequencing electropherograms, are presented in Table S4.

### Statistical analysis

Statistical evaluation of the assay’s performance utilized established epidemiological methods. Sensitivity, specificity, positive predictive value (PPV), negative predictive value (NPV), and kappa statistic, along with 95% confidence intervals (CIs), were calculated via the Wilson score formula. Clinical diagnoses of PTB served as the reference standard for *M. tuberculosis* detection, while non-TB cases were controls. Bacteriologically confirmed NTM cases were deemed positive, with non-cultured cases as controls. ROC analysis assessed overall diagnostic accuracy, represented by AUC-ROC, where an AUC of 0.5 indicates a non-informative classifier and 1 indicates optimal classification. Youden’s index, integrating sensitivity and specificity, was calculated, with values from 0 to 1, where higher values reflect better performance. Data analyses utilized SPSS version 21.0 (IBM Corp., Armonk, NY) and R version 4.2.1 (R Foundation for Statistical Computing, Vienna, Austria), with statistical significance set at *P* < 0.05.

## RESULTS

### Linearity and assay optimization evaluation

**Fig 3:**
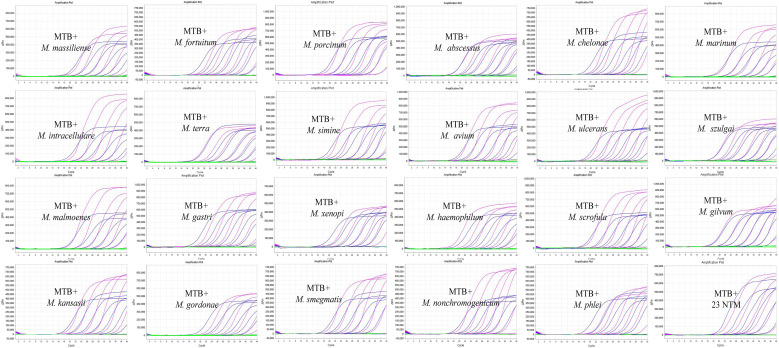
Amplification plots of the multiplex PCR targeting *M. tuberculosis* in combination with individual or all NTM species. The figure demonstrates the amplification curves obtained from the multiplex PCR assay targeting *M. tuberculosis* alongside individual NTM species and a combination of all 23 NTM species. Samples were serially diluted ranging from 10^8^ to 10^2^ copies/mL to evaluate the assay’s performance across different concentrations.

**Fig 4:**
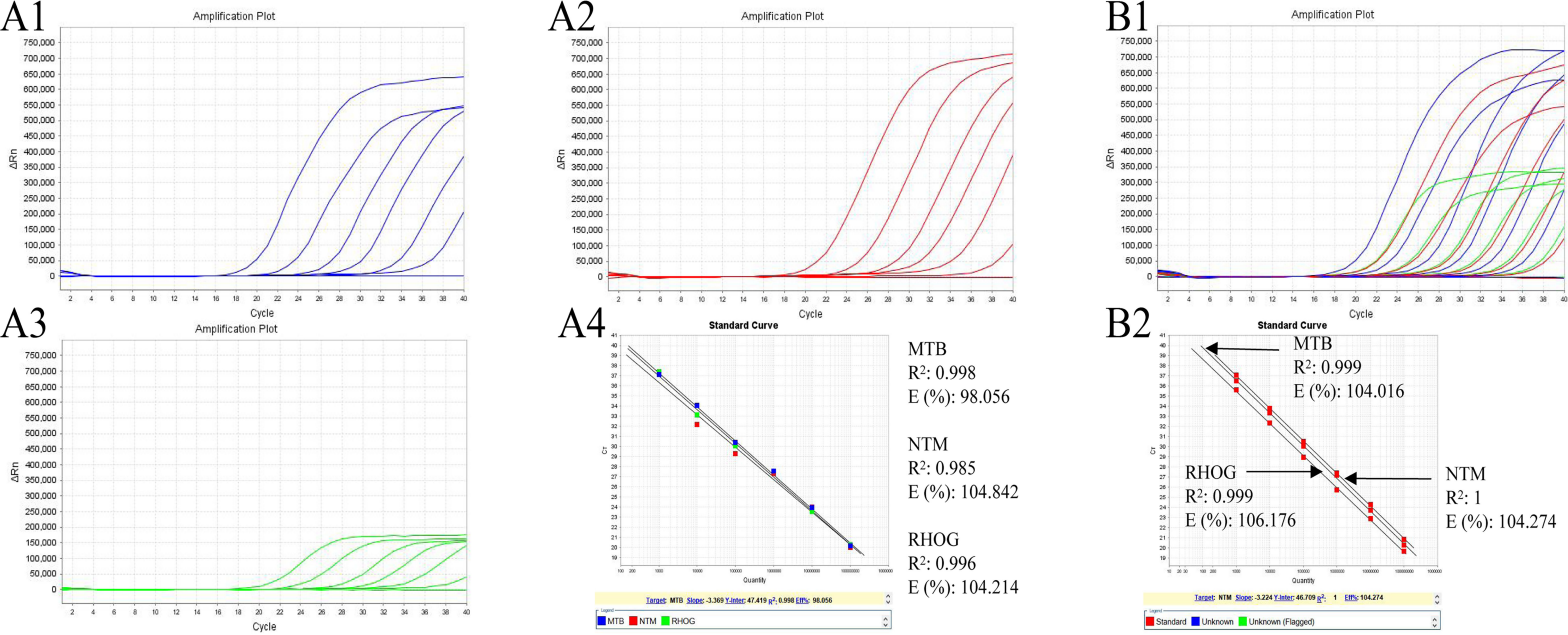
Amplification and standard curves of the multiplex PCR assay for detecting templates from individual or mixed targets at gradient concentrations. This figure provides a comprehensive analysis of the multiplex PCR MTB/NTM assay’s performance across gradient concentrations of individual and mixed nucleic acid targets. (**A1–A3**) The panels display the amplification profiles for 10-fold serial dilutions (10^2^–10^8^ copies/mL) of nucleic acids from *M. tuberculosis*, a mixture of 23 NTM species, and HeLa cells, respectively. (**A4**) The corresponding standard curves for each target. (**B1**) The amplification profiles of mixed nucleic acid samples containing *M. tuberculosis*, 23 NTM species, and HeLa cells at concentrations ranging from 10^2^ to 10^8^ copies/mL. (**B2**) The standard curves for each target in the mixed samples.

Following optimization of primers/probe pairs, thermal cycling conditions, and component concentrations using reference strains, including *M. tuberculosis* and 23 NTM species, singleplex PCR standard curves for *IS6110* (*M. tuberculosis*) and *rpoB* (NTM) demonstrated excellent linearity across a 10-fold dynamic range (10^8^–10^2^ copies/mL), with correlation coefficients (*R*^2^) ranging from 0.993 to 1, and corresponding *E* values from 86.87% to 107.39%. Figure S1 shows robust amplification profiles across varying template concentrations. We expanded the multiplex PCR system to include various nucleic acid templates, including *M. tuberculosis*, a collective of NTM strains, mixtures of *M. tuberculosis* with individual NTM species, and all 23 NTM strains combined with *M. tuberculosis* (amplification curves are illustrated in Fig. 3). The assay exhibited robust amplification for *M. tuberculosis* in the presence of NTM species, demonstrating specificity and sensitivity. Human HeLa cells were incorporated to detect the internal reference gene RHOG. Standard curves from serial dilutions of distinct templates in the multiplex PCR system, either individually or in combination (Fig. 4A1 to A3 and B1), consistently exhibited *R*^2^ values exceeding 0.980 (Fig. 4A4 and B2), particularly with the complete bacterial target spectrum alongside HeLa cells. These curves validate the successful development and optimization of our assay, confirming linearity and minimal interference on amplification efficiency between targets across a wide concentration range, even in complex mixtures.

### Precision and repeatability

Assay precision was evaluated by calculating CV percentages for Ct values from intra-assay and inter-assay experiments. Intra-assay repeatability was validated by testing three concentrations (10^7^, 10^5^ and 10^3^ copies/mL) of DNA from *M. tuberculosis* and 23 individual NTM in triplicates. Table 1 shows intra-assay CVs ranging from 0.05% to 1.18%, indicating exceptional repeatability and measurement precision. Inter-assay verification, repeatedly conducted over three separate days to evaluate the assay’s reproducibility, yielded CVs ranging from 0.08% to 2.57%, confirming the assay’s robustness and consistent results over time. Notably, high precision was maintained even at the LOD of 1,000 copies/mL, demonstrating the assay’s reliability.

**TABLE 1:** Precision evaluation of the multiplex PCR MTB/NTM assay for detection of *M. tuberculosis* and 23 NTM reference strains^*a*^

Target	Concentration (copies/mL)	Intra-assay measurement						Inter-assay measurement					
		Ct of triplicate			Mean	SD	CV%	Mean Ct of three runs			Mean	SD	CV%
*M. tuberculosis*	10^3^	37.44	37.64	37.39	37.49	0.132	0.35	37.12	37.84	37.56	37.51	0.363	0.97
	10^5^	30.41	30.24	30.15	30.27	0.132	0.44	30.14	30.32	30.45	30.30	0.156	0.51
	10^7^	24.32	24.51	24.86	24.56	0.274	1.12	24.5	24.64	24.75	24.63	0.125	0.51
*M. massiliense*	10^3^	37.28	37.15	37.37	37.27	0.111	0.30	37.48	36.87	37.21	37.19	0.306	0.82
	10^5^	30.55	30.72	30.85	30.71	0.150	0.49	30.21	30.98	30.68	30.62	0.388	1.27
	10^7^	24.24	24.56	24.72	24.51	0.244	1.00	24.3	24.48	24.42	24.40	0.092	0.38
*M. intracellulare*	10^3^	36.73	36.77	35.57	36.36	0.682	1.87	36.46	36.58	35.52	36.19	0.580	1.60
	10^5^	29.58	30.02	30.15	29.92	0.299	1.00	30.35	30.32	30.13	30.27	0.119	0.39
	10^7^	23.41	23.48	23.22	23.37	0.135	0.58	23.65	23.54	23.48	23.56	0.086	0.37
*M. malmoenes*	10^3^	37.2	37.12	37.25	37.19	0.066	0.18	37.41	37.33	37.26	37.33	0.075	0.20
	10^5^	30.22	30.39	30.38	30.33	0.095	0.31	30.43	30.42	30.21	30.35	0.124	0.41
	10^7^	23.27	23.35	23.25	23.29	0.053	0.23	23.14	23.58	23.24	23.32	0.231	0.99
*M. kansasii*	10^3^	37.78	37.56	37.38	37.57	0.200	0.53	37.42	37.14	37.24	37.27	0.142	0.38
	10^5^	31.19	31.34	31.28	31.27	0.075	0.24	31.56	31.98	31.22	31.59	0.381	1.21
	10^7^	24.03	24.18	24.12	24.11	0.075	0.31	23.84	24.05	24.18	24.02	0.172	0.71
*M. fortuitum*	10^3^	37.3	37.24	37.1	37.21	0.103	0.28	37.48	37.96	37.88	37.77	0.257	0.68
	10^5^	31.15	31.49	31.13	31.26	0.202	0.65	31.52	31.68	31.43	31.54	0.127	0.40
	10^7^	24.24	24.78	24.69	24.57	0.289	1.18	24.59	24.37	24.64	24.53	0.144	0.59
*M. terra*	10^3^	37.43	37.19	37.29	37.30	0.121	0.32	37.69	37.92	37.41	37.67	0.255	0.68
	10^5^	31.97	31.09	31.67	31.58	0.447	1.42	31.21	31.52	31.55	31.43	0.188	0.60
	10^7^	24.9	24.92	24.92	24.91	0.012	0.05	24.72	24.84	24.92	24.83	0.101	0.41
*M. gastri*	10^3^	37.25	37.91	37.76	37.64	0.346	0.92	37.43	37.98	37.64	37.68	0.278	0.74
	10^5^	30.38	30.43	30.51	30.44	0.066	0.22	30.65	30.67	30.74	30.69	0.047	0.15
	10^7^	23.7	23.62	23.6	23.64	0.053	0.22	23.79	23.63	23.6	23.67	0.102	0.43
*M. gordonae*	10^3^	36.78	37.23	36.99	37.00	0.225	0.61	36.54	37.14	36.81	36.83	0.300	0.82
	10^5^	30.3	30.17	30.53	30.33	0.182	0.60	30.87	30.59	30.62	30.69	0.154	0.50
	10^7^	23.43	23.83	23.52	23.59	0.210	0.89	23.48	23.68	23.64	23.60	0.106	0.45
*M. porcinum*	10^3^	37.52	37.35	37.81	37.56	0.233	0.62	37.31	37.54	37.83	37.56	0.261	0.69
	10^5^	30.63	30.63	30.49	30.58	0.081	0.26	30.88	30.82	30.69	30.80	0.097	0.32
	10^7^	23.85	23.78	23.05	23.56	0.443	1.88	23.72	23.94	24.1	23.92	0.191	0.80
*M. simine*	10^3^	37.37	37.15	37.32	37.28	0.115	0.31	37.77	37.95	37.77	37.83	0.104	0.27
	10^5^	30.86	30.95	30.55	30.79	0.210	0.68	30.12	30.17	30.84	30.38	0.402	1.32
	10^7^	24.13	23.82	23.38	23.78	0.377	1.59	24.51	23.64	23.33	23.83	0.612	2.57
*M. xenopi*	10^3^	37.37	37.15	37.2	37.24	0.115	0.31	36.87	36.98	37.04	36.96	0.086	0.23
	10^5^	30.19	30.39	30.25	30.28	0.103	0.34	30.18	30.22	30.74	30.38	0.312	1.03
	10^7^	23.43	23.86	23.05	23.45	0.405	1.73	23.32	23.35	23.86	23.51	0.303	1.29
*M. gilvum*	10^3^	37.74	37.6	37.73	37.69	0.078	0.21	37.46	37.16	37.21	37.28	0.161	0.43
	10^5^	30.57	30.8	30.78	30.72	0.127	0.41	31.02	31.12	30.75	30.96	0.191	0.62
	10^7^	24.16	24.56	24.26	24.33	0.208	0.86	24.68	24.65	24.25	24.53	0.240	0.98
*M. szulgai*	10^3^	36.91	36.91	36.54	36.79	0.214	0.58	36.75	36.26	36.38	36.46	0.255	0.70
	10^5^	30.14	30.14	30.28	30.19	0.081	0.27	30.35	30.24	30.29	30.29	0.055	0.18
	10^7^	23.54	23.54	23.74	23.61	0.115	0.49	23.86	23.51	23.79	23.72	0.185	0.78
*M. marinum*	10^3^	36.07	36.02	35.68	35.92	0.212	0.59	36.46	36.42	35.86	36.25	0.335	0.93
	10^5^	29.4	29.87	29.73	29.67	0.241	0.81	29.55	29.93	29.86	29.78	0.202	0.68
	10^7^	23.01	23.16	23.69	23.29	0.357	1.53	23.3	23.17	23.74	23.40	0.299	1.28
*M. phlei*	10^3^	37.02	37.35	37.69	37.35	0.335	0.90	37.64	37.18	37.28	37.37	0.242	0.65
	10^5^	30.17	30.22	30.64	30.34	0.258	0.85	30.75	30.24	30.48	30.49	0.255	0.84
	10^7^	23.8	24.13	24.23	24.05	0.225	0.94	23.85	24.08	24.15	24.03	0.157	0.65
*M. scrofula*	10^3^	35.81	36.95	36.02	36.26	0.607	1.67	35.89	36.22	36.64	36.25	0.376	1.04
	10^5^	30.14	30.22	29.68	30.01	0.291	0.97	30.74	30.98	30.66	30.79	0.167	0.54
	10^7^	23.47	23.29	23.46	23.41	0.101	0.43	23.57	23.72	23.78	23.69	0.108	0.46
*M. ulcerans*	10^3^	35.13	35.13	36.18	35.48	0.606	1.71	35.58	35.95	36.31	35.95	0.365	1.02
	10^5^	30.23	30.23	30	30.15	0.133	0.44	30.41	30.65	30.21	30.42	0.220	0.72
	10^7^	23.24	23.24	23.19	23.22	0.029	0.12	23.98	23.82	23.56	23.79	0.212	0.89
*M. chelonae*	10^3^	36.53	36.61	36.2	36.45	0.217	0.60	36.31	36.01	36.38	36.23	0.197	0.54
	10^5^	29.94	30.13	30.27	30.11	0.166	0.55	29.64	30.17	30.85	30.22	0.607	2.01
	10^7^	23.83	23.62	24.1	23.85	0.241	1.01	23.44	23.46	23.65	23.52	0.116	0.49
*M. nonchromogenicum*	10^3^	35.45	36.43	35.79	35.89	0.498	1.39	35.88	35.89	36.36	36.04	0.274	0.76
	10^5^	29.5	29.98	29.22	29.57	0.384	1.30	29.47	29.84	29.81	29.71	0.206	0.69
	10^7^	22.48	23.12	23.06	22.89	0.353	1.54	22.86	22.78	23.15	22.93	0.195	0.85
*M. haemophilum*	10^3^	36.03	35.54	36.1	35.89	0.305	0.85	36.11	36.32	36.02	36.15	0.154	0.43
	10^5^	29.43	29.58	29.28	29.43	0.150	0.51	29.74	29.85	29.34	29.64	0.268	0.91
	10^7^	22.7	22.43	22.49	22.54	0.142	0.63	23.64	23.89	23.72	23.75	0.128	0.54
*M. avium*	10^3^	36.34	36.49	36.69	36.51	0.176	0.48	36.5	36.22	36.66	36.46	0.223	0.61
	10^5^	30.12	29.95	29.42	29.83	0.365	1.22	29.43	29.65	29.48	29.52	0.115	0.39
	10^7^	23.54	23.34	23.29	23.39	0.132	0.57	23.12	22.98	23.37	23.16	0.198	0.85
*M. abscessus*	10^3^	37.84	37.49	37.87	37.73	0.211	0.56	37.42	37.96	37.46	37.61	0.301	0.80
	10^5^	31.08	31.1	31.72	31.30	0.364	1.16	31.32	31.27	31.3	31.30	0.025	0.08
	10^7^	24.88	24.67	24.89	24.81	0.124	0.50	25.16	25.39	25.07	25.21	0.165	0.65
*M. smegmatis*	10^3^	36.46	36.17	36.31	36.31	0.145	0.40	36.15	36.43	36.57	36.38	0.214	0.59
	10^5^	30.09	30.67	30.27	30.34	0.297	0.98	29.71	29.64	29.68	29.68	0.035	0.12
	10^7^	24.05	23.93	23.66	23.88	0.200	0.84	23.78	23.44	23.85	23.69	0.219	0.93

^*a*^ CV, coefficient of variation; SD, standard deviation.

### Analytical sensitivity and specificity evaluation

Standard curves from gradient dilutions of DNA templates revealed that *M. tuberculosis* H37Ra strain was reliably dateable at 1,000 copies/mL, while detection failed at 100 copies/mL, suggesting an estimated lower LOD for *M. tuberculosis* between 250 and 1,000 copies/mL. Similarly, for NTM detection, consistent outcomes were obtained at 1,000 copies/mL but not at 100 copies/mL, implying a comparable LOD for NTM targets. A refined standard curve from 2,000 to 250 copies/mL (Fig. S2) provisionally established an LOD of 1,000 copies/mL for both *M. tuberculosis* and NTM through triplicate measurements. A confirmatory assay with the 24 reference strains repetitively tested 20 times at this defined concentration achieved a 100% detection rate for *M. tuberculosis* and over 95% for 23 NTM strains (Fig. S3), formalizing the LODs of the Multiplex PCR assay at 1,000 copies/mL. Furthermore, a comparative analysis using the Chinese national reference J10 strain of *M. tuberculosis* (CMCC 93009, the only pre-quantified *Mycobacterium* reference strain available in China) corroborated the LOD of 1.000 copies/mL for *M. tuberculosis*, approximately equivalent to 100 bacilli/mL (experimental procedures are detailed in Supplementary Information file 3, including Table S6; Fig. S4).

**Fig 5:**
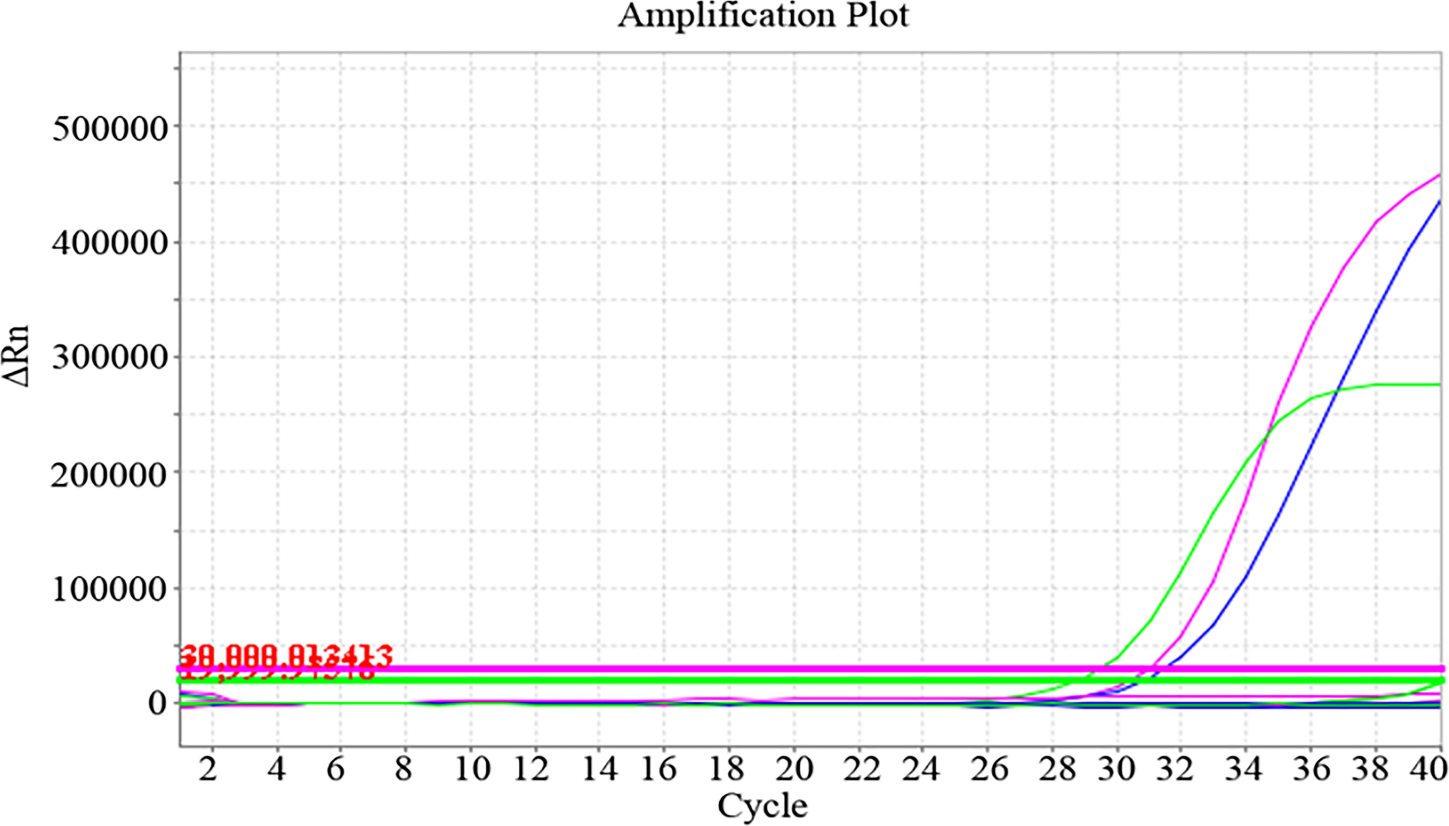
Evaluation of the specificity of the multiplex PCR MTB/NTM assay. This figure evaluates the specificity of the multiplex PCR assay designed to differentiate *M. tuberculosis* from NTM species. The assay was tested at a concentration of 10^5^ copies/mL. Amplification curves are shown for *M. tuberculosis* (FAM channel): Blue curve, NTM species (Texas-Red channel): Red curve, and internal control (CY5 channel): Green curve. No amplification was detected for any of the non-target bacterial strains listed in Table 1, with clear separation of amplification curves for target species from flat lines of non-target strains, confirming the high specificity of the designed primers and probes. The *x*-axis represents PCR cycle number, while the *y*-axis indicates the fluorescence intensity.

Primer specificity was validated using the primer-Blast program, ensuring no non-specific amplification within our assay. Analytical specificity was assessed by simultaneously detecting nucleic acids from a panel of non-target bacteria associated with respiratory infections (Table S1), at 10^5^ copies/mL. Nucleic acid samples from *M. tuberculosis*/NTM (*Mycobacterium massiliense*) and HeLa cells served as positive controls, confirming the absence of non-target bacteria among intended targets. The fluorescence signal from the internal reference gene, RHOG, validated PCR amplification under specific conditions (shown in Fig. 5), demonstrating our assay’s specificity for detecting *M. tuberculosis* and various NTM species in the presence of human DNA, without cross-reactivity to non-mycobacterial strains, highlighting its potential for accurate differential diagnosis of mycobacterial infections in clinical settings.

### Clinical feasibility evaluation

A preliminary diagnostic performance evaluation using 50 sputum samples from patients with culture-confirmed pulmonary TB and AFB smear positivity (32) validated the assay’s diagnostic efficacy for TB, achieving a 100% detection rate for *M. tuberculosis*. Additionally, we retrospectively analyzed clinical isolates of various NTM species co-infected with *M. tuberculosis* as previously identified (10), including five cases of *M. tuberculosis*/*M. intracellulare*, two of *M. tuberculosis*/*M. avium*, one of *M. tuberculosis*/*M. kansasii*, one of *M. tuberculosis*/*M. abscessus*, and one of *M. tuberculosis*/*Mycobacterium malmoense*. All samples in both *M. tuberculosis* and NTM detection channels were accurately reported (Table S5). These findings validate the assay’s detection capabilities for both *M. tuberculosis* and NTM, establishing the foundation for the pilot clinical feasibility study.

**Fig 6:**
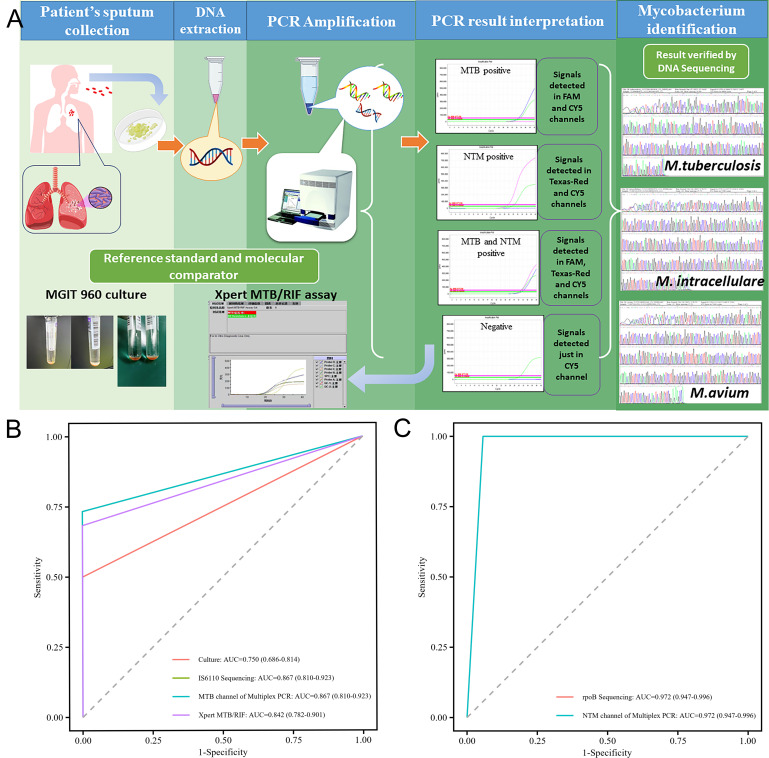
Diagnostic performance evaluation of the Multiplex PCR MTB/NTM assay. (**A**) Diagnostic workflow. This panel graphically illuminates the steps involved in the diagnostic assay, starting from the collection of the patient’s sputum, through DNA extraction, PCR amplification, and concluding with PCR result interpretation. Two main diagnostic tools were compared: the MGIT 960 culture as a reference standard and the Xpert MTB/RIF assay as a molecular comparator. PCR result validation was achieved through DNA sequencing for precise identification of *Mycobacterium* species, such as *M. tuberculosis* and *M. intracellulare*, and positive cases detected by the specific fluorescence channels of the Multiplex PCR MTB/NTM assay are shown by the corresponding amplification curves. (**B**) Receiver operating characteristic (ROC) curves for *M. tuberculosis* detection. ROC curve comparing the sensitivity and specificity of the MTB channel of Multiplex PCR assay, MGIT 960 culture, *IS6110* sequencing, and Xpert MTB/RIF assay for detecting *M. tuberculosis*. Each method’s performance was quantified by the area under the curve (AUC), illustrating their respective diagnostic accuracies. (**C**) ROC curves for NTM detection. ROC curve for the NTM channel of Multiplex PCR assay, compared against *rpoB* sequencing, focusing on the diagnostic accuracy for identifying NTM. The AUC values demonstrate the efficacy of the Multiplex PCR assay in discriminating between *M. tuberculosis* and NTM infections, highlighting its potential utility in clinical settings.

In the pilot study, 96 symptomatic but AFB smear-negative patients were evaluated to assess the assay’s diagnostic accuracy for microscopically indeterminate *M. tuberculosis* infections and potential NTM infections. Figure 6A illustrates the workflow of the Multiplex PCR MTB/NTM assay for pathogen identification in clinical sputum specimens, with PCR results validated through DNA sequencing and compared to the MGIT 960 culture system and Xpert MTB/RIF assay outcomes. Based on comprehensive clinical examination and culture results, 60 patients were diagnosed with PTB, 7 with NTM pulmonary disease, and 29 with LRTI. Our assay detected *M. tuberculosis* in 44 out of 60 PTB samples, exhibiting complete concordance with DNA sequencing, including one *M. intracellulare* co-infection case. The Xpert MTB/RIF assay yielded negative results for three culture-negative cases detected positive by our assay and sequencing. Table 2 summarizes the detection of *M. tuberculosis* and NTM by various methods across the 96 participants, stratified by clinical diagnosis, with summary radiographical findings from CT included. The diagnostic performance characteristics for *M. tuberculosis* detection, using clinical diagnosis as the reference standard, are presented in Table 3. For *M. tuberculosis* in PTB cases, our multiplex assay exhibited a sensitivity of 73.33% (95% CI: 62.1–84.5%), specificity of 100% (95% CI: 100–100%), with a PPV of 100% (95% CI: 100–100%) and NPV of 69.23% (95% CI: 56.7–81.8%). The assay’s AUC (0.867, 95% CI: 0.810–0.923) was comparable to sequencing (0.867) and Xpert MTB/RIF (0.842), and significantly higher than culture (0.750, 95% CI: 68.6–81.4%), highlighting its superior discriminatory power for AFB-smear negative PTB (Fig. 6B).

**TABLE 2:** Molecular, microbiological, and radiographic results from sputum samples from patients with a clinical diagnosis^*a*^

Sputum sample ID	Clinical diagnosis	MGIT960 culture		DNA sequencing		Multiplex PCR		Xpert MTB/RIF	Pulmonary CT findings
		Culture+/MPT64+	Culture+/PNB+	*IS6110* sequencing	*rpoB* sequencing	MTB channel	NTM channel		
1	PTB	＋	−	*M. tuberculosis*	−	＋	−	＋	Nodular lesions, tree-in-bud pattern
2	PTB	−	−	−	−	−	−	−	
3	PTB	−	−	−	−	−	−	−	
4	PTB	−	−	−	−	−	−	−	
5	LRTI	−	−	−	*M. intracellulare*	−	＋	−	Patchy consolidation
6	PTB	＋	−	*M. tuberculosis*	−	＋	−	＋	Nodular lesions, tree-in-bud pattern
7	PTB	−	−	*M. tuberculosis*	−	＋	−	＋	
8	LRTI	−	−	−	−	−	−	−	Pneumonia
9	PTB	−	−	*M. tuberculosis*	−	＋	−	＋	Nodular lesions, tree-in-bud pattern
10	PTB	−	−	−	−	−	−	−	
11	LRTI	−	−	−	−	−	−	−	Pneumonia
12	PTB	＋	−	*M. tuberculosis*	−	＋	−	＋	Nodular lesions
13	LRTI	−	−	−	−	−	−	−	Pneumonia
14	PTB	＋	−	*M. tuberculosis*	−	＋	−	＋	Nodular lesions, tree-in-bud pattern
15	PTB	−	−	*M. tuberculosis*	−	＋	−	＋	
16	PTB	＋	−	*M. tuberculosis*	−	＋	−	＋	
17	NTM	−	＋	−	*M. intracellulare*	−	＋	−	Nodular bronchiectasis
18	PTB	＋	−	*M. tuberculosis*	−	＋	−	＋	Nodular lesions, tree-in-bud pattern
19	LRTI	−	−	−	−	−	−	−	Pneumonia
20	PTB	＋	−	*M. tuberculosis*	−	＋	−	＋	Nodular lesions
21	LRTI	−	−	−	−	−	−	−	Pneumonia
22	PTB	＋	−	MTB	−	＋	−	＋	Nodular lesions, fibrotic cavities
23	LRTI	−	−	−	−	−	−	−	Pneumonia
24	NTM	−	＋	−	*M. avium*	−	＋	−	Nodules
25	PTB	−	−	−	−	−	−	−	Nodular lesions
26	PTB	＋	−	*M. tuberculosis*	−	＋	−	＋	Nodular lesions, fibrotic cavities
27	LRTI	−	−	−	−	−	−	−	Pneumonia
28	PTB	−	−	−	−	−	−	−	Nodular lesions
29	NTM	−	＋	−	*M. abscessus*	−	＋	−	Nodules, thin-walled cavities
30	PTB	−	−	*M. tuberculosis*	−	＋	−	＋	Nodular lesions
31	PTB	＋	−	*M. tuberculosis*	−	＋	−	＋	Nodular lesions, tree-in-bud pattern
32	PTB	＋	−	*M. tuberculosis*	−	＋	−	＋	
33	LRTI	−	−	−	−	−	−	−	Pneumonia
34	PTB	−	−	*M. tuberculosis*	−	＋	−	＋	Nodular lesions
35	LRTI	−	−	−	*M. intracellulare*	−	＋	−	Multiple cavitations
36	PTB	＋	−	*M. tuberculosis*	−	＋	−	＋	Nodular lesions, tree-in-bud pattern
37	LRTI	−	−	−	−	−	−	−	Pneumonia
38	PTB	−	−	−	−	−	−	−	Nodular lesions
39	PTB	−	−	*M. tuberculosis*	−	＋	−	＋	
40	PTB	＋	−	*M. tuberculosis*	−	＋	−	＋	Nodular lesions, tree-in-bud pattern
41	LRTI	−	−	−	−	−	−	−	Pneumonia
42	PTB	＋	−	*M. tuberculosis*	−	＋	−	＋	Nodular lesions, tree-in-bud pattern
43	PTB	＋	−	*M. tuberculosis*	−	＋	−	＋	
44	LRTI	−	−	−	−	−	−	−	Pneumonia
45	PTB	−	−	*M. tuberculosis*	−	＋	−	＋	Nodular lesions
46	LRTI	−	−	−	−	−	−	−	Pneumonia
47	PTB	＋	＋	*M. tuberculosis*	*M. intracellulare*	＋	＋	＋	Nodular lesions, fibrotic cavities
48	PTB	−	−	−	−	−	−	−	Nodular lesions
49	NTM	−	＋	−	*M. intracellulare*	−	＋	−	Nodular bronchiectasis
50	PTB	＋	−	*M. tuberculosis*	−	＋	−	＋	Nodular lesions, tree-in-bud pattern
51	PTB	−	−	*M. tuberculosis*	−	＋	−	−	Nodular lesions
52	PTB	＋	−	*M. tuberculosis*	−	＋	−	＋	Nodular lesions, fibrotic cavities
53	LRTI	−	−	−	−	−	−	−	Pneumonia
54	PTB	＋	−	*M. tuberculosis*	−	＋	−	＋	Nodular lesions, tree-in-bud pattern
55	LRTI	−	−	−	*M. intracellulare*	−	＋	−	Pneumonia
56	PTB	＋	−	*M. tuberculosis*	−	＋	−	＋	Nodular lesions, tree-in-bud pattern
57	LRTI	−	−	−	−	−	−	−	Pneumonia
58	PTB	−	−	*M. tuberculosis*	−	＋	−	−	Nodular lesions
59	LRTI	−	−	−	−	−	−	−	Pneumonia
60	PTB	＋	−	*M. tuberculosis*	−	＋	−	＋	Nodular lesions, tree-in-bud pattern
61	NTM	−	＋	−	*M. fortuitum*	−	＋	−	Nodular bronchiectasis
62	PTB	−	−	−	−	−	−	−	Nodular lesions
63	LRTI	−	−	−	−	−	−	−	Pneumonia
64	PTB	−	−	*M. tuberculosis*	−	＋	−	＋	Nodular lesions
65	PTB	−	−	*M. tuberculosis*	−	＋	−	＋	
66	PTB	−	−	−	−	−	−	−	
67	LRTI	−	−	−	*M. intracellulare*	−	＋	−	Lobar consolidation
68	PTB	＋	−	*M. tuberculosis*	−	＋	−	＋	Nodular lesions, tree-in-bud pattern
69	PTB	−	−	−	−	−	−	−	Nodular lesions
70	PTB	＋	−	*M. tuberculosis*	−	＋	−	＋	Nodular lesions, tree-in-bud pattern
71	LRTI	−	−	−	−	−	−	−	Pneumonia
72	LRTI	−	−	−	−	−	−	−	
73	PTB	＋	−	*M. tuberculosis*	−	＋	−	＋	Nodular lesions, tree-in-bud pattern
74	PTB	−	−	−	−	−	−	−	Nodular lesions
75	PTB	−	−	*M. tuberculosis*	−	＋	−	＋	Nodular lesions
76	LRTI	−	−	−	−	−	−	−	Pneumonia
77	PTB	＋	−	*M. tuberculosis*	−	＋	−	＋	Nodular lesions, tree-in-bud pattern
78	NTM	−	＋	−	*M. avium*	−	＋	−	Nodules
79	PTB	＋	−	*M. tuberculosis*	−	＋	−	＋	Nodular lesions, tree-in-bud pattern
80	PTB	−	−	*M. tuberculosis*	−	＋	−	＋	Nodular lesions
81	LRTI	−	−	−	−	−	−	−	Pneumonia
82	PTB	−	−	−	−	−	−	−	Nodular lesions
83	LRTI	−	−	−	*M. intracellulare*	−	＋	−	Pneumonia
84	PTB	＋	−	*M. tuberculosis*	−	＋	−	＋	Nodular lesions, fibrotic cavities
85	LRTI	−	−	−	−	−	−	−	Pneumonia
86	PTB	＋	−	*M. tuberculosis*	−	＋	−	＋	Nodular lesions, tree-in-bud pattern
87	LRTI	−	−	−	−	−	−	−	Pneumonia
88	PTB	＋	−	*M. tuberculosis*	−	＋	−	＋	Nodular lesions, tree-in-bud pattern
89	LRTI	−	−	−	−	−	−	−	Pneumonia
90	PTB	−	−	−	−	−	−	−	Nodular lesions
91	NTM	−	＋	−	*M. avium*	−	＋	−	Nodules
92	PTB	−	−	−	−	−	−	−	Nodular lesions
93	PTB	−	−	*M. tuberculosis*	−	＋	−	−	Nodular lesions
94	LRTI	−	−	−	−	−	−	−	Pneumonia
95	PTB	＋	−	*M. tuberculosis*	−	＋	−	＋	Nodular lesions, fibrotic cavities
96	PTB	−	−	−	−	−	−	−	Nodular lesions

^*a*^ LRTI, lower respiratory tract infections; MTB, *Mycobacterium tuberculosis*; NTM, non-tuberculous mycobacteria; PNB, *p*-nitro benzoic acid; PTB, pulmonary tuberculosis. + indicates a positive result for the sample using the corresponding detection method, while − indicates a negative result. Based on the MGIT960 liquid bacterial culture method, the determination of *Mycobacterium tuberculosis* culture positivity requires both a positive culture tube report and a positive TB antigen MPT64 immunochromatographic assay. The determination of NTM culture positivity requires both a positive culture tube report and bacterial growth in a medium containing PNB. Due to space limitations in this table, individual CT examination reports for each case cannot be fully listed. Instead, summary diagnostic terms are displayed. Where specific CT findings do not significantly differ between cases, common medical terminology representing shared characteristics is typically selected.

**TABLE 3:** Comparative diagnostic performance of multiplex PCR MTB/NTM assay, DNA sequencing, Xpert MTB/RIF assay and MGIT960 culture for *M. tuberculosis* detection^*a*^

Assay	Results	Diagnosis		Total	Sensitivity (95% CI)	Specificity (95% CI)	PPV (95% CI)	NPV (95% CI)	AUC (95% CI)	Kappa (95% CI)	Youden index
		PTB	Not PTB								
Multiplex PCR MTB channel	Positive	44	0	44	0.733 (0.621–0.845)	1.000 (1.000–1.000)	1.000 (1.000–1.000)	0.692 (0.567–0.818)	0.867 (0.810–0.923)	0.673 (0.535–0.812)	0.733
	Negative	16	36	52							
	Total	60	36	96							
*IS6110* sequencing	Positive	44	0	44	0.733 (0.621–0.845)	1.000 (1.000–1.000)	1.000 (1.000–1.000)	0.692 (0.567–0.818)	0.867 (0.810–0.923)	0.673 (0.535–0.812)	0.733
	Negative	16	36	52							
	Total	60	36	96							
Xpert MTB/RIF	Positive	41	0	41	0.683 (0.566–0.801)	1.000 (1.000–1.000)	1.000 (1.000–1.000)	0.655 (0.529–0.780)	0.842 (0.782–0.901)	0.618 (0.476–0.760)	0.683
	Negative	19	36	55							
	Total	60	36	96							
MGIT960 culture	Positive	30	0	30	0.500 (0.373–0.627)	1.000 (1.000–1.000)	1.000 (1.000–1.000)	0.546 (0.425–0.666)	0.750 (0.686–0.814)	0.429 (0.289–0.568)	0.500
	Negative	30	36	66							
	Total	60	36	96							

^*a*^ This table presents a statistical analysis of the diagnostic performance of various methods for detecting *M. tuberculosis* in clinical samples. Performance indicators such as sensitivity, specificity, positive predictive value (PPV), negative predictive value (NPV), area under the curve (AUC), kappa statistic, and Youden index are detailed, each accompanied by 95% confidence intervals (CIs). These metrics offer a statistical measure of the tests’ accuracy in diagnosing *M. tuberculosis* and NTM infections. The reference standards used were clinical diagnoses for pulmonary TB and NTM lung diseases according to diagnostic criteria from authoritative institutions. TP = true positive; FP = false positive; TN = true negative; FN = false negative. Sensitivity is calculated as TP/(TP + FN), specificity as TN/(TN + FP), PPV as TP/(TP + FP), NPV as TN/(TN + FN), and the Youden index as (sensitivity + specificity − 1).

Among 36 non-PTB samples suspected of NTM infection, 12 tested positive via our assay, with PCR results matching sequencing. This included seven *M. intracellulare*, plus one in a confirmed PTB case, three *M. avium*, one *Mycobacterium fortuitum*, and one *M. abscessus*. Bacteriologically, *M. intracellulare* and *M. avium* are slow-growing, non-pigmented species, while *Mycobacterium fortuitum* and *M. abscessus* are rapidly growing. Integrating clinical presentations, culture results, and the ATS/IDSA criteria, eight cases were confirmed as NTM pneumonia, including the PTB case ultimately diagnosed with PTB/NTM co-infection during follow-up, suggesting five potential false positives, all *M. intracellulare*. For NTM detection, our assay demonstrated 100% sensitivity (95% CI: 100–100%) and 94.33% specificity (95% CI: 89.5–99.2%), with a PPV of 61.53% (95% CI: 35.1–87.9%) and NPV of 100% (95% CI: 100–100%), using clinical diagnosis combined with bacteriological culture as the reference standard (Table 4). Our assay’s calculated AUC was 0.972 (95% CI: 0.947–0.996), identical to sequencing (Fig. 6C). Overall agreement with clinical diagnosis was substantial for both *M. tuberculosis* (kappa = 0.673, 95% CI: 0.535–0.812) and NTM (kappa = 0.735, 95% CI: 0.562–0.953) detection. The Youden index of 0.733 for *M. tuberculosis* detection and 0.943 for NTM identification indicates an optimal sensitivity-specificity balance. These results support the clinical feasibility and diagnostic accuracy of the assay in challenging smear-negative specimens.

**TABLE 4:** Comparative diagnostic performance of multiplex PCR MTB/NTM assay, DNA sequencing, Xpert MTB/RIF assay and MGIT960 culture for *M. tuberculosis* detection^*a*^

Assay	Results	Diagnosis		Total	Sensitivity (95% CI)	Specificity (95% CI)	PPV (95% CI)	NPV (95% CI)	AUC (95% CI)	Kappa (95% CI)	Youden index
		NTM	Not NTM								
Multiplex PCR NTM channel	Positive	8	5	13	1.000 (1.000-1,000)	0.943 (0.895-0.992)	0.615 (0.351-0.880)	1.000 (1.000-1,000)	0.972 (0.947-0.996)	0.735 (0.562-0.953)	0.943
	Negative	0	83	83							
	Total	7	89	96							
*rpoB* sequencing	Positive	8	5	13	1.000 (1.000-1,000)	0.943 (0.895-0.992)	0.615 (0.351-0.880)	1.000 (1.000-1,000)	0.972 (0.947-0.996)	0.735 (0.562-0.953)	0.943
	Negative	0	83	83							
	Total	8	88	96							

^*a*^ This table presents a statistical analysis of the diagnostic performance of various methods for detecting NTM in clinical samples. Performance indicators such as sensitivity, specificity, positive predictive value (PPV), negative predictive value (NPV), area under the curve (AUC), kappa statistic, and Youden index are detailed, each accompanied by 95% confidence intervals (CIs). These metrics offer a statistical measure of the tests’ accuracy in diagnosing *M. tuberculosis* and NTM infections. The reference standards used were clinical diagnoses for pulmonary TB and NTM lung diseases according to diagnostic criteria from authoritative institutions. TP = true positive; FP = false positive; TN = true negative; FN = false negative. Sensitivity is calculated as TP/(TP + FN), specificity as TN/(TN + FP), PPV as TP/(TP + FP), NPV as TN/(TN + FN), and the Youden index as (sensitivity + specificity − 1).

## DISCUSSION

Differentiating *M. tuberculosis* from increasingly prevalent NTM remains a critical challenge, particularly in high-burden regions like China, and especially for smear-negative cases where traditional diagnostics seem insuperable (40). The WHO’s 2024 Bacterial Priority Pathogens List underscores rifampicin-resistant *M. tuberculosis* as a critical threat, highlighting the urgency for innovative diagnostics to prevent misdiagnosis leading to inappropriate treatment and weakened TB control efforts (41). Our multiplex TaqMan PCR assay directly addresses this gap by simultaneously detecting and differentiating *M. tuberculosis* and NTM from a single sample. This approach aligns with China’s TB management framework and the WHO’s TPPs for next-generation diagnostics and aims to enhance molecular capabilities in peripheral settings, facilitating rapid, accurate, and affordable diagnosis crucial for appropriate patient management and TB elimination goals.

Analytically, the assay demonstrated a dynamic range (10^8^–10^3^ copies/mL), minimal intra-assay (0.05–1.18%), and inter-assay (0.08–2.57%) variability, and a consistent LOD of 1,000 copies/mL for both *M. tuberculosis* and NTM. Clinically evaluated in 96 smear-negative patients, it achieved 73.33% sensitivity and 100% specificity for *M. tuberculosis* detection, and 100% sensitivity and 94.33% specificity for NTM identification, using clinical diagnosis and culture as reference standard. Among 60 PTB cases, it identified *M. tuberculosis* in 14 cases missed by culture, highlighting its value in challenging samples to conventional methods (14). Notably, of 36 non-PTB cases, despite seven clinically diagnosed NTM pneumonia, our assay detected NTM in five patients ultimately diagnosed with LRTI and identified a clinically significant *M. tuberculosis/M. intracellulare* co-infection that was initially missed, underscoring both its sensitivity to low bacterial loads and the complexity of interpreting NTM presence, which requires careful clinical correlation (13).

A key innovation is the carefully optimized selection of *IS6110* for *M. tuberculosis* and *rpoB* for NTM detection within a single-tube multiplex format. While other assays utilized targets like the internal transcribed spacer (ITS) region and the *devR* gene for MTBC and *IS1311* or *16S rRNA* for NTM, or multiple gene combinations like *IS6110* and *senX3-regX3* genes, *IS 1311* and *DT1*, or *ITS* and *16S rRNA* (21–23, 42), or even complicated targets like *rv0577*, *RD9*, and *mtbk_20670* for *M. tuberculosis*, and *IS1331*, *DT1*, and *mkan_rs12360* for NTM (25), potentially increasing complexity, our approach focuses on established markers. The *IS6110* gene offers robust sensitivity and specificity for MTBC due to its transposable and multi-copy nature while minimizing cross-reactivity (30, 43). The *rpoB* gene provides sufficient polymorphism for differentiating mycobacterial species while retaining conserved regions for species-specific identification (31). We deliberately avoid the widely used *16S rRNA* gene due to its limited ability to reliably differentiate *M. tuberculosis* from diverse NTM species (44). Our oligonucleotide design minimizes interference and non-specific amplification between the targets.

Technically, the assay employs distinct fluorescent TaqMan probes for simultaneous *M. tuberculosis* and NTM detection and differentiation in a single reaction, building upon our multiplexing experience (32, 39). This one-step approach offers advantages in workflow efficiency and reduced contamination risk compared to multi-step protocols that generalize two-round PCR for distinguishing MTBC from NTM (24). Inclusion of the human RHOG verifies sample adequacy and guards against false negatives due to inhibition or poor sample quality (39). Rigorous optimization combined with hot-start Taq DNA polymerase and UDG enzyme ensures reproducibility and minimizes non-specific amplification and carryover contamination (23), contributing to the robust performance observed during evaluation.

Addressing the diagnostic challenge of differentiating *M. tuberculosis* from NTM in smear-negative sputum (14), where Ziehl-Neelsen smear microscopy reported an 8.6% smear-positive rate (38), we conducted this prospective pilot study specifically in this difficult-to-diagnose population. Unlike evaluations often limited to cultured strains or pre-confirmed samples (22, 23, 25), our approach assessed real-world clinical utility. Despite limitations inherent in our pilot study, conceptual rigor was maintained through adherence to diagnostic criteria from Chinese TB guidelines (33), WHO recommendations (34), and ATS/IDSA guidelines (13), along with sample size scientifically justified, establishing a robust framework for positive case definition. Methodologically, the use of multiple comparators (culture, sequencing, Xpert MTB/RIF), double-blind, and 3-month clinical follow-up enhances the relevance of our findings compared to purely laboratory-based validations (22, 25).

Our Multiplex PCR MTB/NTM assay demonstrated strong diagnostic potential, particularly for *M. tuberculosis* detection in this challenging smear-negative cohort, aligning with WHO recommendations for improved molecular diagnostics (18, 20). Its *M. tuberculosis* diagnostic performance (73.33% sensitivity, 100% specificity) compared favorably with reported Xpert MTB/RIF performance in similar population (61–67% sensitivity, 99% specificity) (45). Furthermore, despite potential *IS6110* copy number variations (46–48), our validated LOD of 1,000 copies/mL (approximately 100 bacilli/mL) proved robust and is comparable to Xpert MTB/RIF’s LOD of 131 CFU/mL (45). While overall performance for *M. tuberculosis* falls within the range of other multiplex assays, such as reported by Sarro et al. (83.3% sensitivity, 96.6% specificity) (21) and Kim et al. (87.5% sensitivity, 99.6% specificity) (24), direct comparison is nuanced as prior studies often included smear-positive samples. For NTM identification, our assay achieved excellent performance (100% sensitivity, 94.33% specificity) within the encountered spectrum. However, reflecting the pilot nature of our study and common challenges in NTM clinical research, despite retrospective validation with stored isolates of co-infections, we finally validated the assay’s capacity to identify six NTM species: *M. intracellulare*, *M. avium*, *Mycobacterium fortuitum*, *M. kansasii*, *Mycobacterium malmoense*, and *M. abscessus* out of the 23 targeted, due to limited clinical encounters with diverse NTM cases. This limitation, also observed in comparable studies (e.g., 5 MAC cases in Sarro et al., 40 NTM samples yielding 53.3% sensitivity and 99.9% specificity in Kim et al.), hinders definitive cross-assay comparisons for NTM performance but highlights the need for broader validation. Nonetheless, the robust *M. tuberculosis* detection in smear-negative samples underscores the assay’s clinical utility.

Beyond performance, our assay offers practical advantages over more complex laboratory-developed tests (21, 22, 24, 25). Its simplified design enables a sample-to-result time within 3 h with reduced cross-contamination risk, making it beneficial in peripheral laboratories with limited resources (14, 18). Demonstrated user-friendliness facilitates large-scale implementation. Notably, stringent cost control allows a projected price of $5.00 per test, promoting accessibility in regions burdened by *M. tuberculosis* and NTM infections (8, 49) and adhering to WHO TPP affordability criteria (19). Combined with our complementary multiplex assay for rifampicin and isoniazid resistance (32), this offers a comprehensive and cost-effective molecular diagnostic strategy (≤$15.00 total) attractive for public health policymakers.

This study unavoidably presents limitations affecting generalizability. The modest sample size (*n* = 96) and single-center design restrict statistical power and applicability across diverse settings (35, 36). Future multi-center studies should involve larger cohorts from diverse geographical regions over extended periods, with appropriate patient stratification to ensure representativeness (50). Testing only one sputum specimen per patient, while pragmatic, might underestimate maximal sensitivity. Testing multiple specimens collected at different times would be a future investigation. The prospective enrollment, targeting TB-suggestive symptoms, limited the number and diversity of NTM cases encountered (12, 13), preventing full validation of the assay’s capacity for all 23 targeted species. Targeted retrospective studies utilizing characterized NTM isolates from specialized institutions are needed to fully evaluate NTM detection capabilities. Finally, while promising, our assay should be integrated into, not replace, comprehensive clinical evaluation, including clinical, radiological, and microbiological results (13, 33, 34). Relying solely on our may risk misdiagnosis or overdiagnosis (14). Future studies should assess its optimal position with diagnostic algorithms.

### Conclusion

We developed and validated a multiplex real-time PCR assay for simultaneous detection and differentiation of *M. tuberculosis* and NTM in smear-negative sputum samples. Its robust analytical performance, high diagnostic accuracy, efficient workflow, and affordability make it a promising tool for rapid mycobacterial infections in resource-limited settings. Our assay significantly advances TB and NTM infection management by facilitating early diagnosis and timely treatment, thereby improving patient outcomes, optimizing healthcare resources, and supporting the WHO’s End TB Strategy objectives.

## Data Availability

The article and its supplemental files encompass all pertinent data. Raw experimental data, including export files of PCR reactions, and derived data supporting the findings of this study can be available from the corresponding authors X.-W.J. and Z.C. upon reasonable request.

## SUPPLEMENTAL MATERIAL

Supplemental material spectrum.02316-24-s0001.docx Tables S1 to S6; Fig. S1 to S4.

ASM does not own the copyrights to Supplemental Material that may be linked to, or accessed through, an article. The authors have granted ASM a non-exclusive, world-wide license to publish the Supplemental Material files. Please contact the corresponding author directly for reuse.

## References

[1] Glickman MS, Jacobs WR Jr. 2001. Microbial pathogenesis of Mycobacterium tuberculosis: dawn of a discipline. Cell 104:477–485. doi:10.1016/s0092-8674(01)00236-7 11239406

[2] World Health Organization. 2024. Global tuberculosis report 2024. World Health Organization, Geneva.

[3] Velayati AA, Farnia P, Mozafari M, Mirsaeidi M. 2015. Nontuberculous mycobacteria isolation from clinical and environmental samples in Iran: twenty years of surveillance. Biomed Res Int 2015:254285. doi:10.1155/2015/254285 26180788 PMC4477424

[4] Martín-Casabona N, Bahrmand AR, Bennedsen J, Thomsen VO, Curcio M, Fauville-Dufaux M, Feldman K, Havelkova M, Katila ML, Köksalan K, Pereira MF, Rodrigues F, Pfyffer GE, Portaels F, Urgell JR, Rüsch-Gerdes S, Tortoli E, Vincent V, Watt B, Spanish Group for Non-Tuberculosis Mycobacteria. 2004. Non-tuberculous mycobacteria: patterns of isolation. A multi-country retrospective survey. Int J Tuberc Lung Dis 8:1186–1193. 15527150

[5] Hoefsloot W, van Ingen J, Andrejak C, Angeby K, Bauriaud R, Bemer P, Beylis N, Boeree MJ, Cacho J, Chihota V, et al.. 2013. The geographic diversity of nontuberculous mycobacteria isolated from pulmonary samples: an NTM-NET collaborative study. Eur Respir J 42:1604–1613. doi:10.1183/09031936.00149212 23598956

[6] Thomson RM, NTM working group at Queensland TB Control Centre and Queensland Mycobacterial Reference Laboratory. 2010. Changing epidemiology of pulmonary nontuberculous mycobacteria infections. Emerg Infect Dis 16:1576–1583. doi:10.3201/eid1610.091201 20875283 PMC3294381

[7] Huang JJ, Li YX, Zhao Y, Yang WH, Xiao M, Kudinha T, Xu YC. 2020. Prevalence of nontuberculous mycobacteria in a tertiary hospital in Beijing, China, January 2013 to December 2018. BMC Microbiol 20:158. doi:10.1186/s12866-020-01840-5 32532202 PMC7291475

[8] Yu X, Liu P, Liu G, Zhao L, Hu Y, Wei G, Luo J, Huang H. 2016. The prevalence of non-tuberculous mycobacterial infections in mainland China: systematic review and meta-analysis. J Infect 73:558–567. doi:10.1016/j.jinf.2016.08.020 27717784

[9] Wang L, Zhang H, Ruan Y, Chin DP, Xia Y, Cheng S, Chen M, Zhao Y, Jiang S, Du X, He G, Li J, Wang S, Chen W, Xu C, Huang F, Liu X, Wang Y. 2014. Tuberculosis prevalence in China, 1990-2010; a longitudinal analysis of national survey data. Lancet 383:2057–2064. doi:10.1016/S0140-6736(13)62639-2 24650955

[10] Huang M, Tan Y, Zhang X, Wang Y, Su B, Xue Z, Wang J, Pang Y. 2022. Effect of mixed infections with Mycobacterium tuberculosis and nontuberculous mycobacteria on diagnosis of multidrug-resistant tuberculosis: a retrospective multicentre study in China. Infect Drug Resist 15:157–166. doi:10.2147/IDR.S341817 35082503 PMC8786360

[11] Prevots DR, Marras TK. 2015. Epidemiology of human pulmonary infection with nontuberculous mycobacteria: a review. Clin Chest Med 36:13–34. doi:10.1016/j.ccm.2014.10.002 25676516 PMC4332564

[12] Gomathy NS, Padmapriyadarsini C, Silambuchelvi K, Nabila A, Tamizhselvan M, Banurekha VV, Lavanya J, Chandrasekar C. 2019. Profile of patients with pulmonary non-tuberculous mycobacterial disease mimicking pulmonary tuberculosis. Indian J Tuberc 66:461–467. doi:10.1016/j.ijtb.2019.04.013 31813432

[13] Daley CL, Iaccarino JM, Lange C, Cambau E, Wallace RJ Jr, Andrejak C, Böttger EC, Brozek J, Griffith DE, Guglielmetti L, Huitt GA, Knight SL, Leitman P, Marras TK, Olivier KN, Santin M, Stout JE, Tortoli E, van Ingen J, Wagner D, Winthrop KL. 2020. Treatment of nontuberculous mycobacterial pulmonary disease: an official ATS/ERS/ESCMID/IDSA clinical practice guideline. Eur Respir J 56:2000535. doi:10.1183/13993003.00535-2020 32636299 PMC8375621

[14] Siddiqi K, Lambert ML, Walley J. 2003. Clinical diagnosis of smear-negative pulmonary tuberculosis in low-income countries: the current evidence. Lancet Infect Dis 3:288–296. doi:10.1016/s1473-3099(03)00609-1 12726978

[15] Provoost J, Valour F, Gamondes D, Roux S, Freymond N, Perrot E, Souquet PJ, Kiakouama-Maleka L, Chidiac C, Lina G, Dumitrescu O, Sénéchal A, Ader F. 2018. A retrospective study of factors associated with treatment decision for nontuberculous mycobacterial lung disease in adults without altered systemic immunity. BMC Infect Dis 18:659. doi:10.1186/s12879-018-3559-x 30547753 PMC6295085

[16] Horan KL, Cangelosi GA. 2017. Drug resistance of non-tuberculous mycobacteria, p 1061–1071. In Mayers DL, Sobel JD, Ouellette M, Kaye KS, Marchaim D (ed), Antimicrobial drug resistance: clinical and epidemiological aspects. Vol. 2. Springer International Publishing, Cham.

[17] Griffith DE, Aksamit T, Brown-Elliott BA, Catanzaro A, Daley C, Gordin F, Holland SM, Horsburgh R, Huitt G, Iademarco MF, Iseman M, Olivier K, Ruoss S, von Reyn CF, Wallace RJ Jr, Winthrop K, ATS Mycobacterial Diseases Subcommittee, American Thoracic Society, Infectious Disease Society of America. 2007. An official ATS/IDSA statement: diagnosis, treatment, and prevention of nontuberculous mycobacterial diseases. Am J Respir Crit Care Med 175:367–416. doi:10.1164/rccm.200604-571ST 17277290

[18] Cazabon D, Suresh A, Oghor C, Qin ZZ, Kik SV, Denkinger CM, Pai M. 2017. Implementation of Xpert MTB/RIF in 22 high tuberculosis burden countries: are we making progress? Eur Respir J 50:1700918. doi:10.1183/13993003.00918-2017 28860268

[19] Schumacher SG, Wells WA, Nicol MP, Steingart KR, Theron G, Dorman SE, Pai M, Churchyard G, Scott L, Stevens W, Nabeta P, Alland D, Weyer K, Denkinger CM, Gilpin C. 2019. Guidance for studies evaluating the accuracy of sputum-based tests to diagnose tuberculosis. J Infect Dis 220:S99–S107. doi:10.1093/infdis/jiz258 31593597 PMC6782025

[20] World Health Organization. 2014. High priority target product profiles for new tuberculosis diagnostics: report of a consensus meeting, 28-29 April 2014, Geneva, Switzerland. World Health Organization, Geneva.

[21] Sarro YDS, Butzler MA, Sanogo F, Kodio O, Tolofoudie M, Goumane MS, Baya B, Diabate S, Diallo IB, Daniogo D, et al.. 2021. Development and clinical evaluation of a new multiplex PCR assay for a simultaneous diagnosis of tuberculous and nontuberculous mycobacteria. EBioMedicine 70:103527. doi:10.1016/j.ebiom.2021.103527 34391092 PMC8365364

[22] Sevilla IA, Molina E, Elguezabal N, Pérez V, Garrido JM, Juste RA. 2015. Detection of mycobacteria, Mycobacterium avium subspecies, and Mycobacterium tuberculosis complex by a novel tetraplex real-time PCR assay. J Clin Microbiol 53:930–940. doi:10.1128/JCM.03168-14 25588660 PMC4390653

[23] Shrestha NK, Tuohy MJ, Hall GS, Reischl U, Gordon SM, Procop GW. 2003. Detection and differentiation of Mycobacterium tuberculosis and nontuberculous mycobacterial isolates by real-time PCR. J Clin Microbiol 41:5121–5126. doi:10.1128/JCM.41.11.5121-5126.2003 14605148 PMC262464

[24] Kim JU, Ryu DS, Cha CH, Park SH. 2018. Paradigm for diagnosing mycobacterial disease: direct detection and differentiation of Mycobacterium tuberculosis complex and non-tuberculous mycobacteria in clinical specimens using multiplex real-time PCR. J Clin Pathol 71:774–780. doi:10.1136/jclinpath-2017-204945 29559518

[25] Chae H, Han SJ, Kim SY, Ki CS, Huh HJ, Yong D, Koh WJ, Shin SJ. 2017. Development of a one-step multiplex PCR assay for differential detection of major Mycobacterium species. J Clin Microbiol 55:2736–2751. doi:10.1128/JCM.00549-17 28659320 PMC5648710

[26] Scott L, David A, Govender L, Furrer J, Rakgokong M, Waja Z, Martinson N, Eisenberg G, Marlowe E, Stevens W. 2020. Performance of the Roche cobas MTB assay for the molecular diagnosis of pulmonary tuberculosis in a high HIV burden setting. J Mol Diagn 22:1225–1237. doi:10.1016/j.jmoldx.2020.06.018 32745613 PMC7527868

[27] de Vos M, Derendinger B, Dolby T, Simpson J, van Helden PD, Rice JE, Wangh LJ, Theron G, Warren RM. 2018. Diagnostic accuracy and utility of FluoroType MTBDR, a new molecular assay for multidrug-resistant tuberculosis. J Clin Microbiol 56:e00531-18. doi:10.1128/JCM.00531-18 29976588 PMC6113470

[28] Park KS, Kim JY, Lee JW, Hwang YY, Jeon K, Koh WJ, Ki CS, Lee NY. 2013. Comparison of the Xpert MTB/RIF and Cobas TaqMan MTB assays for detection of Mycobacterium tuberculosis in respiratory specimens. J Clin Microbiol 51:3225–3227. doi:10.1128/JCM.01335-13 23863563 PMC3811628

[29] Bicmen C, Gunduz AT, Coskun M, Senol G, Cirak AK, Ozsoz A. 2011. Molecular detection and identification of Mycobacterium tuberculosis complex and four clinically important nontuberculous mycobacterial species in smear-negative clinical samples by the genotype mycobacteria direct test. J Clin Microbiol 49:2874–2878. doi:10.1128/JCM.00612-11 21653780 PMC3147717

[30] McEvoy CRE, Falmer AA, Gey van Pittius NC, Victor TC, van Helden PD, Warren RM. 2007. The role of IS6110 in the evolution of Mycobacterium tuberculosis. Tuberculosis (Edinb) 87:393–404. doi:10.1016/j.tube.2007.05.010 17627889

[31] Lee H, Bang HE, Bai GH, Cho SN. 2003. Novel polymorphic region of the rpoB gene containing Mycobacterium species-specific sequences and its use in identification of mycobacteria. J Clin Microbiol 41:2213–2218. doi:10.1128/JCM.41.5.2213-2218.2003 12734283 PMC154678

[32] Xie L, Zhu X-Y, Xu L, Xu X-X, Ruan Z-F, Huang M-X, Chen L, Jiang X-W. 2024. Accurate and affordable detection of rifampicin and isoniazid resistance in Tuberculosis sputum specimens by multiplex PCR-multiple probes melting analysis. Infection 52:2371–2398. doi:10.1007/s15010-024-02295-w 38884858 PMC11621165

[33] National Center of Medical Quality Control for Respiratory Diseases, Tuberculosis Branch of Chinese Medical Association, Tuberculosis Control Branch of Chinese Antituberculosis Association, China-Japan Friendship Hospital. 2024. Clinical practice guidelines for early detection of pulmonary tuberculosis in general medical facilities. J Tuberc Lung Dis 5:1–14. doi:10.19982/j.issn.1000-6621.20230428

[34] World Health Organization. 2022. WHO consolidated guidelines on tuberculosis: module 3: diagnosis: tests for TB infection. World Health Organization, Geneva.

[35] Billingham SAM, Whitehead AL, Julious SA. 2013. An audit of sample sizes for pilot and feasibility trials being undertaken in the United Kingdom registered in the United Kingdom Clinical Research Network database. BMC Med Res Methodol 13:104. doi:10.1186/1471-2288-13-104 23961782 PMC3765378

[36] Hajian-Tilaki K. 2014. Sample size estimation in diagnostic test studies of biomedical informatics. J Biomed Inform 48:193–204. doi:10.1016/j.jbi.2014.02.013 24582925

[37] World Health Organization. 2004. Edited by A. D. Harries, D. Maher, S. Graham, and C. Gilks. TB/HIV: a clinical manual. 2nd ed. World Health Organization, Geneva.

[38] Xia H, Song YY, Zhao B, Kam KM, O’Brien RJ, Zhang ZY, Sohn H, Wang W, Zhao YL. 2013. Multicentre evaluation of Ziehl-Neelsen and light-emitting diode fluorescence microscopy in China. Int J Tuberc Lung Dis 17:107–112. doi:10.5588/ijtld.12.0184 23232010

[39] Jiang XW, Huang TS, Xie L, Chen SZ, Wang SD, Huang ZW, Li XY, Ling WP. 2022. Development of a diagnostic assay by three-tube multiplex real-time PCR for simultaneous detection of nine microorganisms causing acute respiratory infections. Sci Rep 12:13306. doi:10.1038/s41598-022-15543-6 35922526 PMC9427838

[40] Chin DP. 2021. The COVID-19 pandemic and elimination of tuberculosis in China. China CDC Wkly 3:260–264. doi:10.46234/ccdcw2021.069 34594862 PMC8392955

[41] World Health Organization. 2024. WHO bacterial priority pathogens list, 2024: bacterial pathogens of public health importance, to guide research, development and strategies to prevent and control antimicrobial resistance. World Health Organization, Geneva.

[42] Singh K, Kumari R, Tripathi R, Gupta S, Anupurba S. 2020. Detection of clinically important non tuberculous mycobacteria (NTM) from pulmonary samples through one-step multiplex PCR assay. BMC Microbiol 20:267. doi:10.1186/s12866-020-01952-y 32847517 PMC7448335

[43] Hellyer TJ, DesJardin LE, Assaf MK, Bates JH, Cave MD, Eisenach KD. 1996. Specificity of IS6110-based amplification assays for Mycobacterium tuberculosis complex. J Clin Microbiol 34:2843–2846. doi:10.1128/jcm.34.11.2843-2846.1996 8897197 PMC229418

[44] Sambo F, Finotello F, Lavezzo E, Baruzzo G, Masi G, Peta E, Falda M, Toppo S, Barzon L, Di Camillo B. 2018. Optimizing PCR primers targeting the bacterial 16S ribosomal RNA gene. BMC Bioinformatics 19:343. doi:10.1186/s12859-018-2360-6 30268091 PMC6162885

[45] Steingart KR, Schiller I, Horne DJ, Pai M, Boehme CC, Dendukuri N. 2014. Xpert MTB/RIF assay for pulmonary tuberculosis and rifampicin resistance in adults. Cochrane Database Syst Rev 2014:CD009593. doi:10.1002/14651858.CD009593.pub3 24448973 PMC4470349

[46] Gutiérrez MC, Vincent V, Aubert D, Bizet J, Gaillot O, Lebrun L, Le Pendeven C, Le Pennec MP, Mathieu D, Offredo C, Pangon B, Pierre-Audigier C. 1998. Molecular fingerprinting of Mycobacterium tuberculosis and risk factors for tuberculosis transmission in Paris, France, and surrounding area. J Clin Microbiol 36:486–492. doi:10.1128/JCM.36.2.486-492.1998 9466764 PMC104565

[47] Mokrousov I, Narvskaya O, Vyazovaya A, Otten T, Jiao WW, Gomes LL, Suffys PN, Shen AD, Vishnevsky B. 2012. Russian “successful” clone B0/W148 of Mycobacterium tuberculosis Beijing genotype: a multiplex PCR assay for rapid detection and global screening. J Clin Microbiol 50:3757–3759. doi:10.1128/JCM.02001-12 22933595 PMC3486266

[48] Gagneux S, DeRiemer K, Van T, Kato-Maeda M, de Jong BC, Narayanan S, Nicol M, Niemann S, Kremer K, Gutierrez MC, Hilty M, Hopewell PC, Small PM. 2006. Variable host-pathogen compatibility in Mycobacterium tuberculosis. Proc Natl Acad Sci USA 103:2869–2873. doi:10.1073/pnas.0511240103 16477032 PMC1413851

[49] World Health Organization. 2022. Global tuberculosis report 2022. World Health Organization, Geneva.

[50] Bossuyt PM, Reitsma JB, Bruns DE, Gatsonis CA, Glasziou PP, Irwig L, Lijmer JG, Moher D, Rennie D, de Vet HCW, Kressel HY, Rifai N, Golub RM, Altman DG, Hooft L, Korevaar DA, Cohen JF, STARD Group. 2015. STARD 2015: an updated list of essential items for reporting diagnostic accuracy studies. BMJ 351:h5527. doi:10.1136/bmj.h5527 26511519 PMC4623764

