# Supplementary material for: Simultaneous detection and differentiation of Mycobacterium tuberculosis and nontuberculous mycobacteria in smear-negative sputum by a multiplex PCR assay: a clinical feasibility study
Source: Microbiol Spectr. 2025 May 30;13(7):e02316-24. doi: 10.1128/spectrum.02316-24 (PMC12210955; doi:10.1128/spectrum.02316-24)
Supplement: Supplemental material — Tables S1 to S6; Fig. S1 to S4. [file spectrum.02316-24-s0001.docx]

## **Supplementary Materials for**

## **Simultaneous detection and differentiation of *Mycobacterium tuberculosis* and Nontuberculous mycobacteria in smear-negative sputum by a multiplex PCR assay: a clinical feasibility study**

Supplementary Text

The Supplementary Information document includes three additional files. The first additional file outlines the general methods and materials information that complements the main text of the manuscript. The second additional file describes the additional result table and figures that need to be included. The third additional file focuses on the validation of the limit of detection (LOD) of the *IS6110* primers-probe set in the Multiplex PCR MTB/NTM assay, which comprises independent sections for methods, results, and discussion.

**Supplementary information**

**Additional file 1: Supplementary information to the main text of the Methods and Materials section**

**Sample size calculation**

The formula for our sample size calculation is expressed as:

$$n=\frac{{Z_{\frac{\left( 1-\alpha\right)}{2}}}^{2}\times V\left( \hat{AUC} \right)}{d^{2}}$$

where $Z_{\left( 1-\alpha\right)/2}$ is the critical value of the normal distribution for a significance level α, d is the permissible error margin, and $V\left( \hat{AUC} \right)$ represents the variance of AUC, calculated using the formula: $V\left( \hat{AUC} \right)=\left( 0.0099\times e^{-a^{2}/2} \right)\times\left( 6a^{2}+16 \right)$, where $a=\varphi^{-1}\left( AUC \right)\times1.414, and \varphi^{-1}$is the inverse of the standard cumulative normal distribution, e is the natural logarithm base. For an expected AUC of 0.85, with α set at 0.05 and d set at 0.1, the calculated $Z_{\left( 1-\alpha\right)/2}$value leads to 1.96. Applying these values in our sample size formula yields: n=38. Considering the potential for a 10% dropout rate, as commonly accounted for in clinical studies, we adjusted the number of subjects per group from 38 to 42. Therefore, each group (cases and controls) needed to enrol about 42 patients, leading to a total of 84 participants required for robust study outcomes. This sample size calculation ensures that the study is statistically powered to reliably evaluate the efficacy of the Multiplex PCR MTB/NTM assay in the targeted clinical scenario.

**Bacterial strain and clinical isolates**

**Table S1: List of bacterial strains and human HeLa cells used as reference controls for development and evaluation of the Multiplex PCR MTB/NTM assay.**

| Study Number | Assay Target | Species | Supplier’s Number | Supplier | Description |
| --- | --- | --- | --- | --- | --- |
| 1 | MTB | *Mycobacterium tuberculosis* | ATCC 25177 | American Type Culture Collection | Cultured bacterial strains, supplied in frozen powder phase |
| 2 | NTM | *Mycobacterium abscessus* | ATCC 19977 |  |  |
| 3 |  | *Mycobacterium fortuitum* | ATCC 6841 |  |  |
| 4 |  | *Mycobacterium gordonae* | ATCC 14470 |  |  |
| 5 |  | *Mycobacterium haemophilum* | ATCC 29548 |  |  |
| 6 |  | *Mycobacterium intracellulare* | ATCC 13950 |  |  |
| 7 |  | *Mycobacterium kansasii* | ATCC 12478 |  |  |
| 8 |  | *Mycobacterium ulcerans* | ATCC 19423 |  |  |
| 9 |  | *Mycobacterium marinum* | ATCC 927 |  |  |
| 10 |  | *Mycobacterium nonchromogenicum* | ATCC 19530 |  |  |
| 11 |  | *Mycobacterium phlei* | ATCC 111758 |  |  |
| 12 |  | *Mycobacterium scrofula* | ATCC19981 |  |  |
| 13 |  | *Mycolicibacterium smegmatis* | ATCC 19420 |  |  |
| 14 |  | *Mycolicibacterium szulgai* | ATCC 35799 |  |  |
| 15 |  | *Mycobacterium malmoenes* | ATCC 29571 |  |  |
| 16 |  | *Mycobacterium gastri* | ATCC 15754 |  |  |
| 17 |  | *Mycobacterium terra* | ATCC 15755 |  |  |
| 18 |  | *Mycobacterium gilvum* | ATCC 43909 |  |  |
| 19 |  | *Mycobacterium porcinum* | CCUG 37674 | Culture Collection University of Gothenburg, Göteborg, Sweden | Cultured bacterial strains, supplied in frozen powder phase |
| 20 |  | *Mycobacterium chelonae* | CCUG 47445 |  |  |
| 21 |  | *Mycolicibacterium simine* | CCUG 29114 |  |  |
| 22 |  | *Mycobacterium xenopi* | CCUG 28011 |  |  |
| 23 |  | *Mycobacterium avium* | CCUG 20992 |  |  |
| 24 |  | *Mycobacterium massiliense* | CCUG 48898T |  |  |
| 25 | Bacterial species unrelated to the assay targets, used for evaluation of assay’s specificity | *Nocardia brasiliensis* |  | National Institute for Food and Drug Control, Beijing, China | Inactivated clinical isolates, supplied in frozen liquid phase |
| 26 |  | *Corynebacterium pekinense* |  |  |  |
| 27 |  | *Streptococcus pneumoniae* |  |  |  |
| 28 |  | *Legionella pneumophila* |  |  |  |
| 29 |  | *Bordetella pertussis* |  |  |  |
| 30 |  | *Chlamydia pneumoniae* | ATCC VR-1360 | American Type Culture Collection | Cultured bacterial strains, supplied in frozen powder phase |
| 31 |  | *Staphylococcus epidermidis* | ATCC 12228 |  |  |
| 32 |  | *Cryptococcus neoformans* | ATCC 32609 |  |  |
| 33 |  | *Rhodococcus equi* | ATCC 33701 |  |  |
| 34 |  | *Haemophilus influenzae* | GDMCC 1.961 | Guangdong Microbial Culture Collection Center, Guangzhou, China | Cultured bacterial strains, supplied in frozen powder phase |
| 35 |  | *Nocardia asteroides* | GDMCC 4.141 |  |  |
| 36 |  | *Staphylococcus aureus* | CMCC 26003 | National Center for Medical Culture Collections, Beijing, China | Cultured bacterial strains, supplied in frozen powder phase |
| 37 |  | *Escherichia coli* | CMCC 44102 |  |  |
| 38 |  | *Pseudomonas aeruginosa* | CMCC 10104 |  |  |
| 39 |  | *Candida albicans* | CMCC 98001 |  |  |
| 40 | Internal control | *HeLa* | ATCC CCL-2 | American Type Culture Collection | Human cell line, supplied in frozen powder phase |

**Design of primer-probe pairs for the Multiplex PCR MTB/NTM assay**

**Table S2: Primer and probe sequences for the Multiplex PCR MTB/NTM assay.**

| Target gene | Species targeted by primer sequences | Primer/probe name and sequences | Working concentration (nM) | Annealing temp (℃) | Amplicon size (bp) |
| --- | --- | --- | --- | --- | --- |
| *IS6110* (MTB) | *M. tuberculosis, M. bovis, M. afticanum, M. microti, M. caprae* | MF: 5’- AGGTACCCGCCGGAGCTG -3’ | 300 | 63 | 161 |
|  |  | MR: 5’- GCATCGACCTGCGCCTG-3’ | 300 | 61.2 |  |
|  |  | MP: 5’-FAM-CGCACCGCCCGCRCACG -BHQ-1-3’ | 100 | 70.2 |  |
| *rpoB* (NTM) | *M. abscessus, M. fortuitum, M. chelonae,  M. intracellulare, M. nonchromogenicum, M. phlei, M. xenopi, M. smegmatis, M. scrofula,  M. malmoenes, M. szulgai, M. simine, M. ulcerans, M. marinum, M. porcinum, M. massiliense,  M. haemophilum* | NF1: 5’- GAGGACATGCCGTTCCTGC -3’ | 200 | 60.2 | 121 |
|  | *M. gordonae* | NF2: 5’- GAGGACATGCCGTTCATGC -3’ | 100 | 58.6 |  |
|  | *M. terra* | NF3: 5’- GAGGACATGCCCTTCCTGC -3’ | 100 | 59.2 |  |
|  | *M. avium* | NF4: 5’- GGCAAGATCCTGCCGCAG -3’ | 100 | 61.4 | 139 |
|  | *M. kansasii, M. gastri* | NF5: 5’- GGCAAGATCCTGGCGCAA -3’ | 100 | 61.8 |  |
|  | *M. gilvum* | NF6: 5’-GAGGACATGCCGTTCCTCC -3’ | 100 | 59 |  |
|  | Universal NTM | NR: 5’-GGATCTGRCCGATGTTCATMCG-3’ | 300 | 62.3 |  |
|  | Universal NTM | NP: 5’-Texas-Red- CCGGTSGACATCATCCT -MGB -3’ | 100 | 73 |  |
| RHOG | Human cells | RF: 5’- ACTACAGCAACTGCACCCACGA -3’ | 200 | 63 | 80 |
|  |  | RR: 5’- TAGCAGATGAGCAGGCACGTCTT -3’ | 200 | 64 |  |
|  |  | RP: 5’- CY5-AGAGCATCAAGTGCGTGGTGGTGG - BHQ-1 -3’ | 100 | 70.1 |  |

**Absolute quantification by droplet digital PCR**

Reaction mixtures were prepared with 11 µl ddPCR supermix for probes (supplied by Bio-Rad Laboratories, Hercules, CA), ddPCR primers and probes (final concentration of 450 nM and 200 nM, respectively), and 4 µl template nucleic acids in a final volume of 22 µl. Each reaction was loaded into the sample well of an eight-well droplet cartridge together with 70 µl of droplet generation oil (Bio-Rad). Following droplet formation in a QX200 droplet generator (Bio-Rad), the droplets were transferred to a 96-well PCR plate, heat-sealed with foil, and subject to amplification using a Longene^®^ A200 Gradient Thermal Cycler (Longene Scientific Instrument, Hangzhou, China). The PCR parameters were as follows: initial denaturation at 95℃ for 10 min, followed by 40 cycles of 95℃ for 50 s and 58℃ for 1 min, and a final extension step at 72℃ for 5 min. The PCR plate was subsequently scanned on a QX200 droplet reader (Bio-Rad) and the copies/µl of each target per well were analysed with QuantaSoft software version 1.7 (Bio-Rad). Droplet positivity was determined by fluorescence intensity, with only droplets above a minimum threshold of fluorescence amplitude considered positive. The copy number per milliliter of each reference strain were determined absolutely by converting the concentration tube to the actual sample concentration.

**Table S3: Primer sequences used in DNA sequencing and droplet digital PCR.**

| Application | Target microorganism | Target gene | Primer name and sequences (5’- 3’) | Working concentration (nM) | Annealing temp (℃) | Amplicon size (bp) |
| --- | --- | --- | --- | --- | --- | --- |
| Digital droplet PCR | MTB | *IS6110* | MDF: 5’- AGGTACCCGCCGGAGCTG -3’ | 300 | 63.0 | 161 |
|  |  |  | MDR: 5’- GCATCGACCTGCGCCTG-3’ | 300 | 61.2 |  |
|  |  |  | MDP: 5’-FAM-CGCACCGCCCGCRCACG -BHQ-1-3’ | 100 | 70.2 |  |
|  | NTM | *rpoB* | NDF: 5’- GACATCTACCGCAAGCTGCG-3’ | 150 | 61.7 | 212 |
|  |  |  | NDR: 5’-TACTCGATGGTGGCGACGAC-3’ | 300 | 61.5 |  |
|  |  |  | NDP: 5’-FAM-CTGTTCTTCAAGGAGAAGCG-MGB -3’ | 100 | 69.2 |  |
| DNA sequencing | MTB | *IS6110* | MSF: 5’- GAAAGACGTTATCCACCATACGGA -3’ | 300 | 60.1 | 359 |
|  |  |  | MSR: 5’-GAGTCCGGAGACTCTCTGATCTGA -3’ | 300 | 60 |  |
|  | NTM | *rpoB* | NSF: 5’- GGCTTCTCCGAGATCATGATG-3’ | 300 | 58.3 | 486 |
|  |  |  | NSR: 5’-CGGCGTGATCGCCTCG-3’ | 300 | 62.1 |  |
|  |  |  | HaeSF⃰: 5’- GTCGGGGTTTACGTCGCC -3’ | 300 | 59.9 | 160 |
|  |  |  | HaeSR⃰: 5’- CCCCATGGGTGTTGAGGATG -3’ | 300 | 61.9 |  |

⃰ Due to the failure of the NSF and NSR primer pairs to amplify the *M. haemophilum* template, we have added a new set of specific primers, HaeSF and HaeSR, to our NTM sequencing system. The products amplified by the two primer pairs differ in size and can be distinguished on 1% agarose gel electrophoresis.

**DNA sequencing**

The sequencing was performed at Sangon Biotech (Shanghai, China) on an Applied Biosystems 3730xl DNA Analyzer (Thermo-Fisher Scientific, Foster City, CA), following the contractor’s standard procedure. Prior to sequencing, preliminary amplification of nucleic acids from the 96 clinical samples was carried out in the MBETRC laboratory. Conventional PCR was performed under the following thermal cycling conditions: 50°C for 2 min (1 cycle), 95°C for 15 min (1 cycle), followed by 40 cycles of 95°C for 15 s and 55°C for 45 s.

**Table S4: Outcomes of sequence alignment and** **representative electropherograms.**

| Mycobacterium species | Genbank accession number of the most similar species | Representative Electropherogram |
| --- | --- | --- |
| *Mycobacterium tuberculosis* | CP133601.1 | 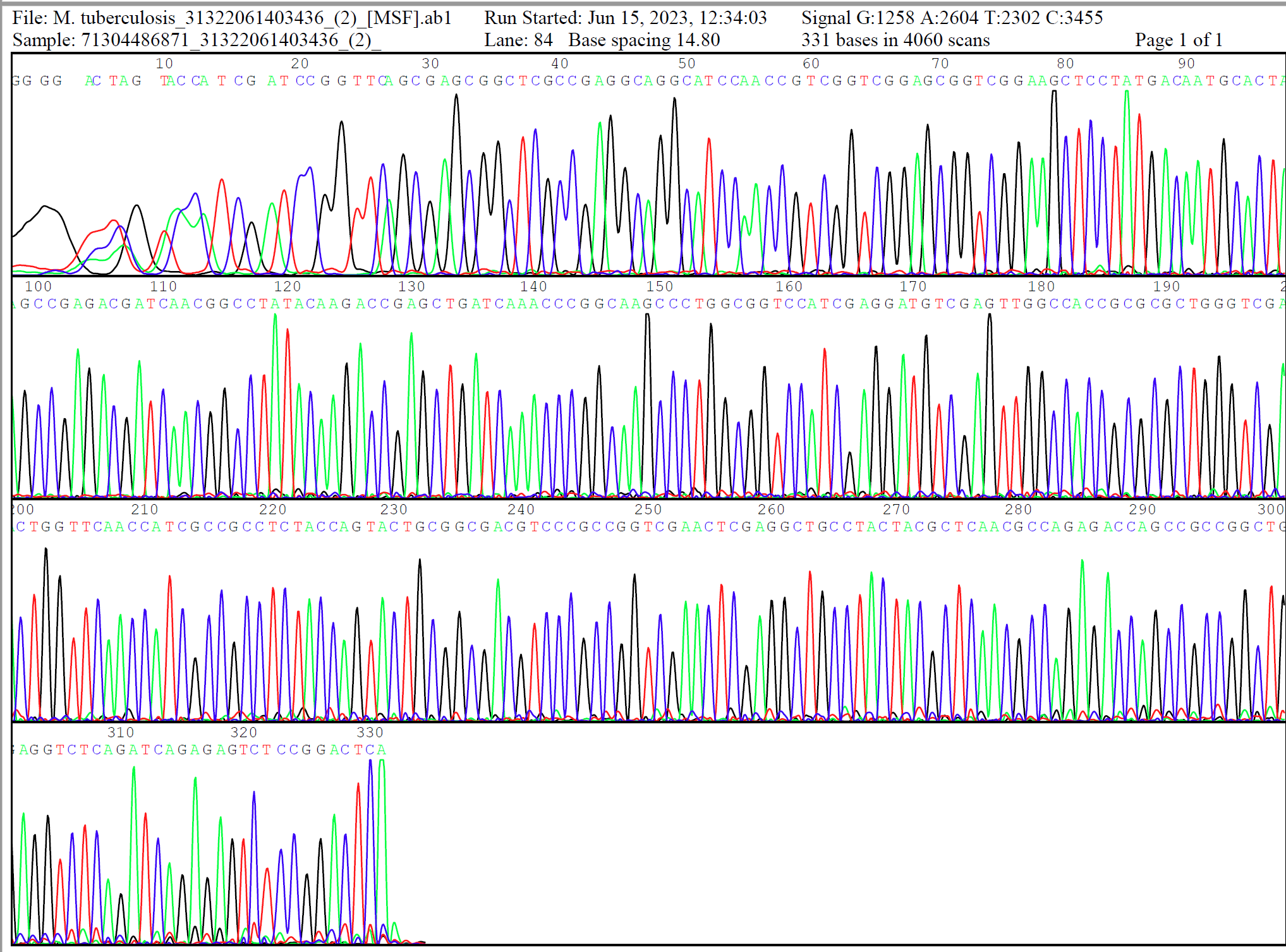 |
| *Mycobacterium intracellulare* | [CP023149.1](https://www.ncbi.nlm.nih.gov/nucleotide/CP023149.1?report=genbank&log$=nuclalign&blast_rank=2&RID=UWM9B2GJ016) | 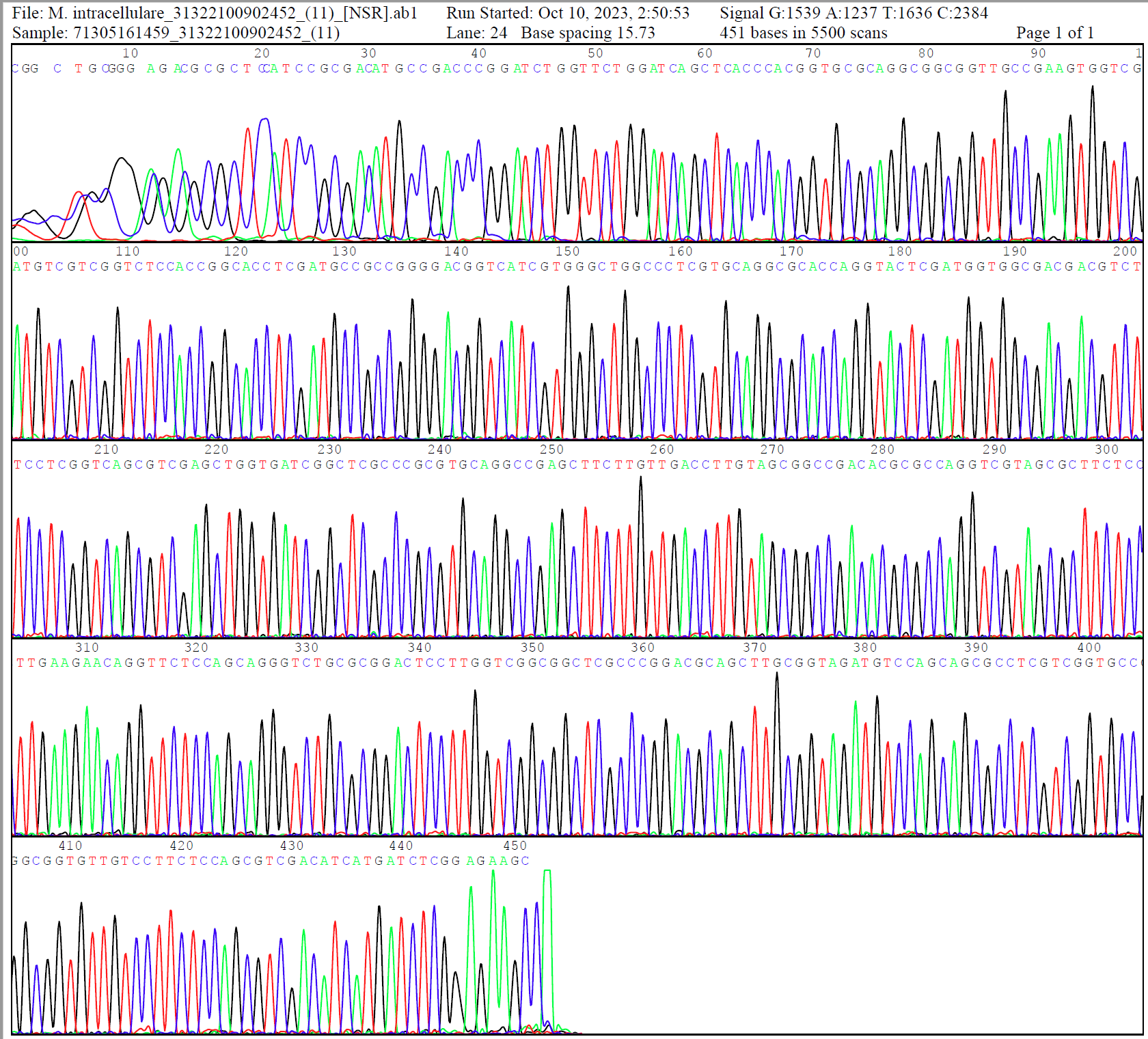 |
| *Mycobacterium avium* | [CP046507.1](https://www.ncbi.nlm.nih.gov/nucleotide/CP046507.1?report=genbank&log$=nucltop&blast_rank=1&RID=UWJM4CM101N) | 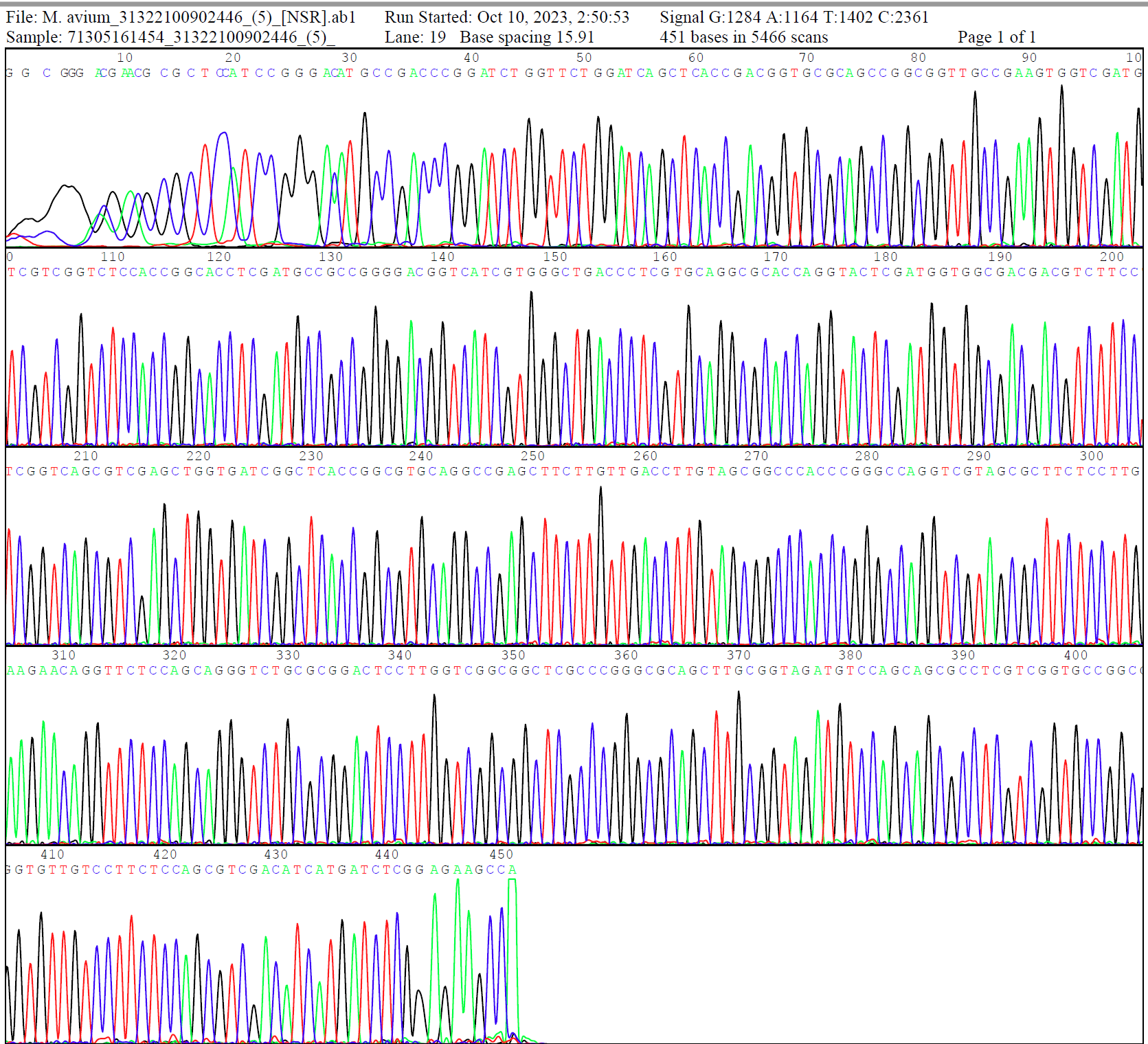 |
| *Mycobacterium fortuitum* | [CP014258.1](https://www.ncbi.nlm.nih.gov/nucleotide/CP014258.1?report=genbank&log$=nuclalign&blast_rank=1&RID=UWMHV31C013) | 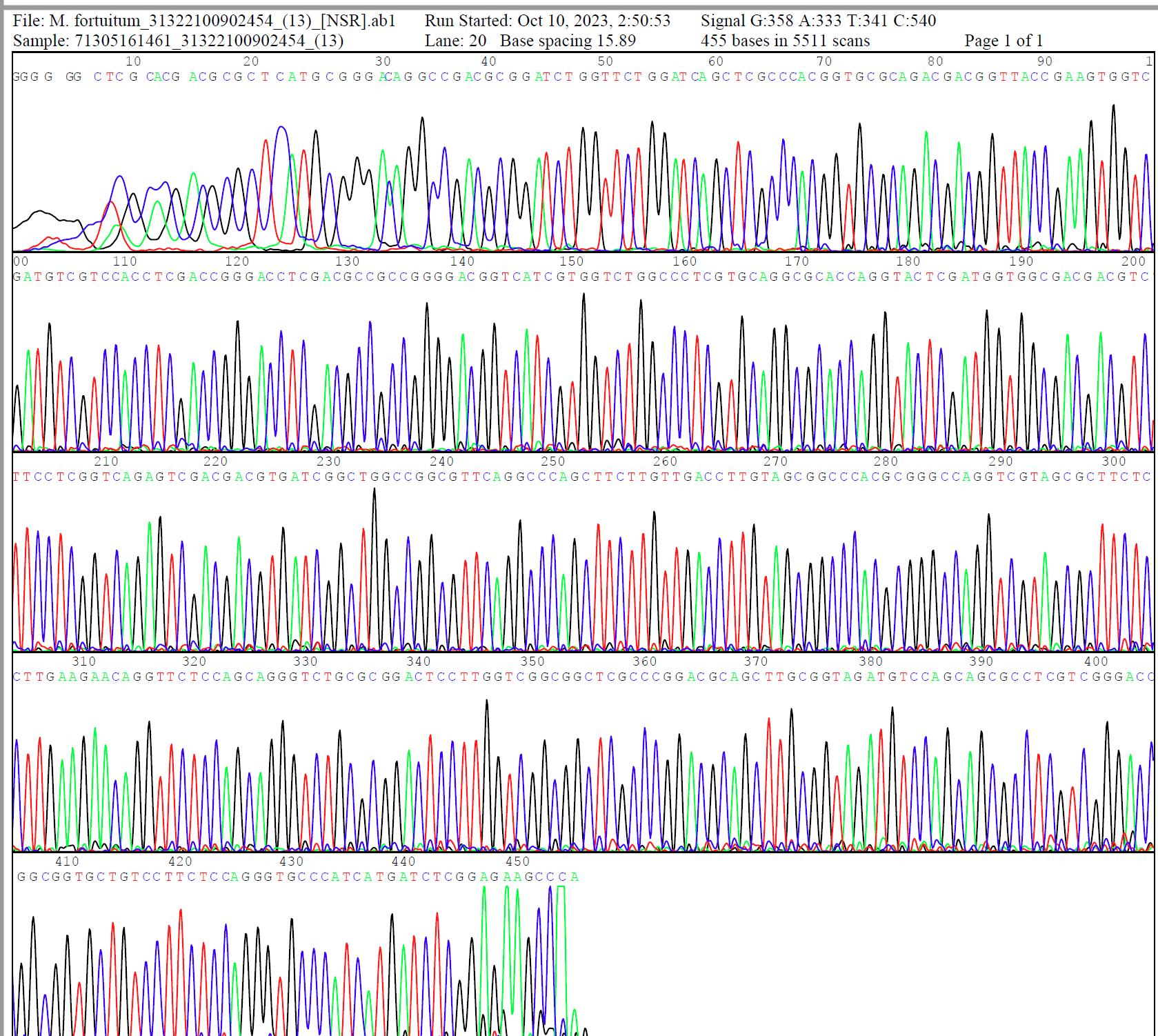 |
| *Mycobacteroides abscessus* | [CP050978.1](https://www.ncbi.nlm.nih.gov/nucleotide/CP050978.1?report=genbank&log$=nuclalign&blast_rank=1&RID=VHNZ754E013) | 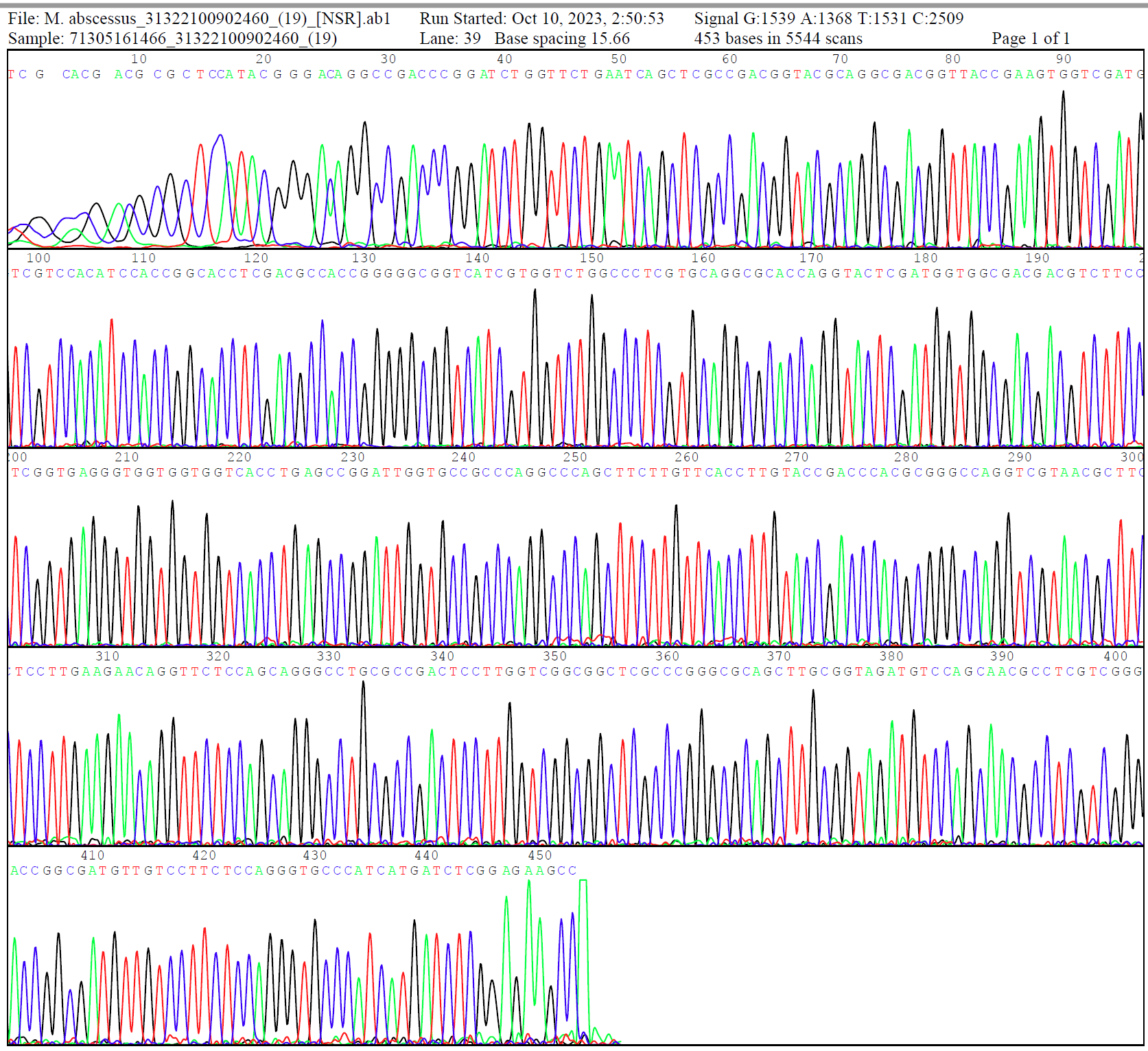 |

Note: DNA sequencing of nucleic acids extracted from 96 clinical specimens was performed based on Sanger’s method. Sanger Sequencing was performed by Sangon Biotech and the raw data output as fluorescent peak trace chromatograms was supplied by this contractor as well. BLAST search was carried out to verify sequences producing significant alignments. Electropherogram Data was exported by using the Chromas software version 2.6.

**Additional file 2: Supplementary results**

**Table S5: Outcomes of preliminary and retrospective diagnostic performance evaluation experiments.**

| Experimental Assessment | Sample Type | Collaborator | Number of Sample | Detection Target | Multiplex PCR assay Detection Rate in Detection Channel | | Comments |
| --- | --- | --- | --- | --- | --- | --- | --- |
|  |  |  |  |  | MTB | NTM |  |
| Preliminary Diagnostic Performance Evaluation | Sputum Specimen | Chaoshan Hospital of Jinan University | 50 | *M. tuberculosis* | 100% |  | Validated the diagnostic efficacy of the assay for detecting TB |
| Retrospective Diagnostic Performance Validation | Clinical Isolates (*M. tuberculosis/*NTM co-infection) | Fuzhou Pulmonary Hospital (FPH) | 5 | *M. tuberculosis* /*Mycobacterium intracellulare* | 100% | 100% | All samples correctly reported as NTM co-infected with *M. tuberculosis* |
|  |  |  | 2 | *M. tuberculosis* /*Mycobacterium avium* | 100% | 100% |  |
|  |  |  | 1 | *M. tuberculosis* /*Mycobacterium kansasii* | 100% | 100% |  |
|  |  |  | 1 | *M. tuberculosis* /*Mycobacterium abscessus* | 100% | 100% |  |
|  |  |  | 1 | *M. tuberculosis* /*Mycobacterium malmoense* | 100% | 100% |  |

**
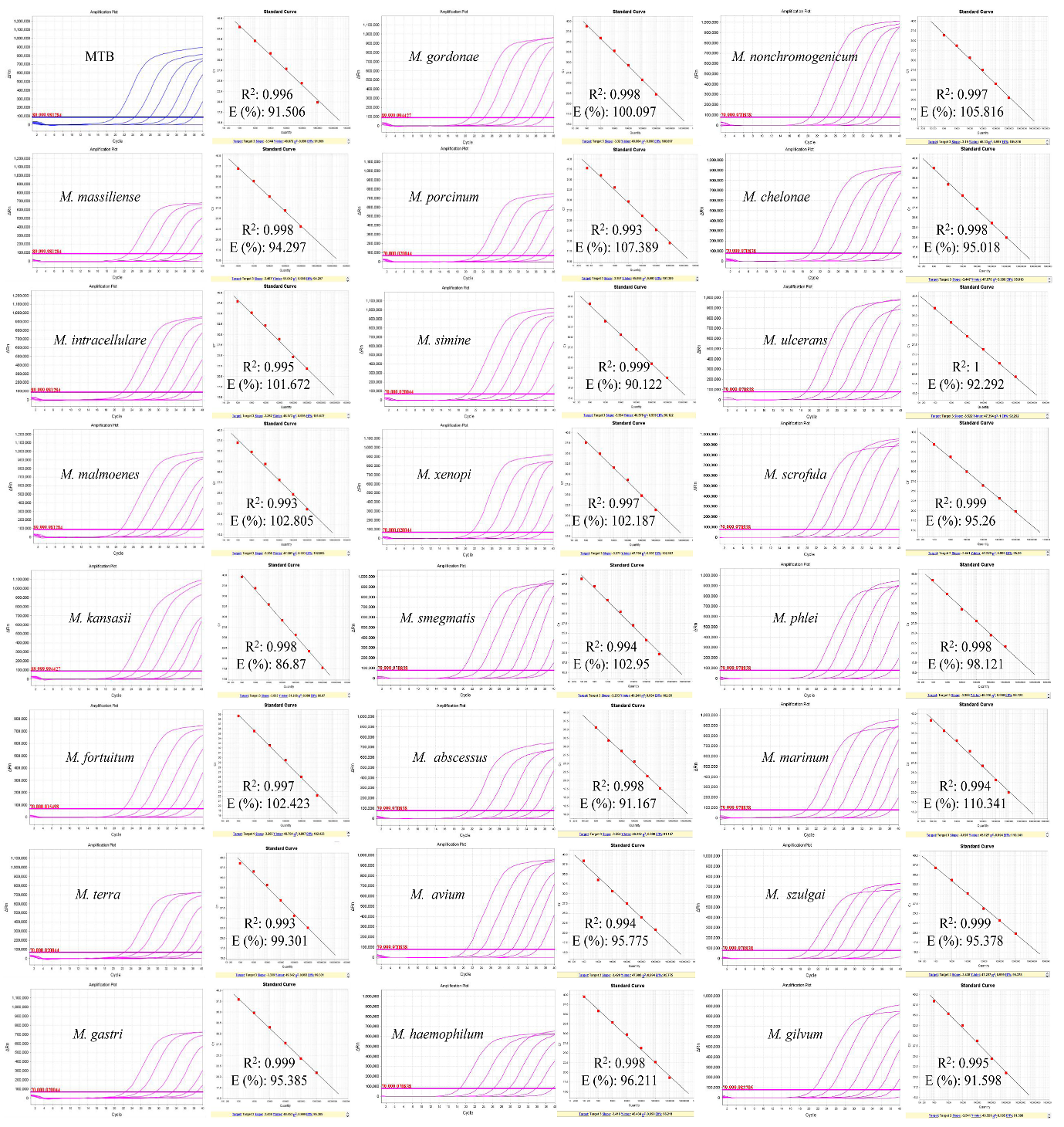
**

**Figure S1: Standard curves from single-plex real-time PCR assays targeting *M. tuberculosis* and 23 NTM species.** Each panel displays the amplification plots and corresponding standard curve generated by a single-plex PCR assay using serially diluted DNA samples (10^2^-10^8^ copies/ml) from either *M. tuberculosis* or one of 23 NTM species. Amplification curves demonstrate robust detection of the target species across the tested concentration range. The linearity and efficiency of each assay is reflected by the R^2^ and E (%) values, respectively, which are annotated on the standard curve in each panel. R^2^ values exceeded 0.99 for all assays, indicating excellent linearity. PCR efficiency ranged from 86.87% to 110.30% across the different assays. Collectively, these data establish the analytic performance of the component single-plex PCR assays.


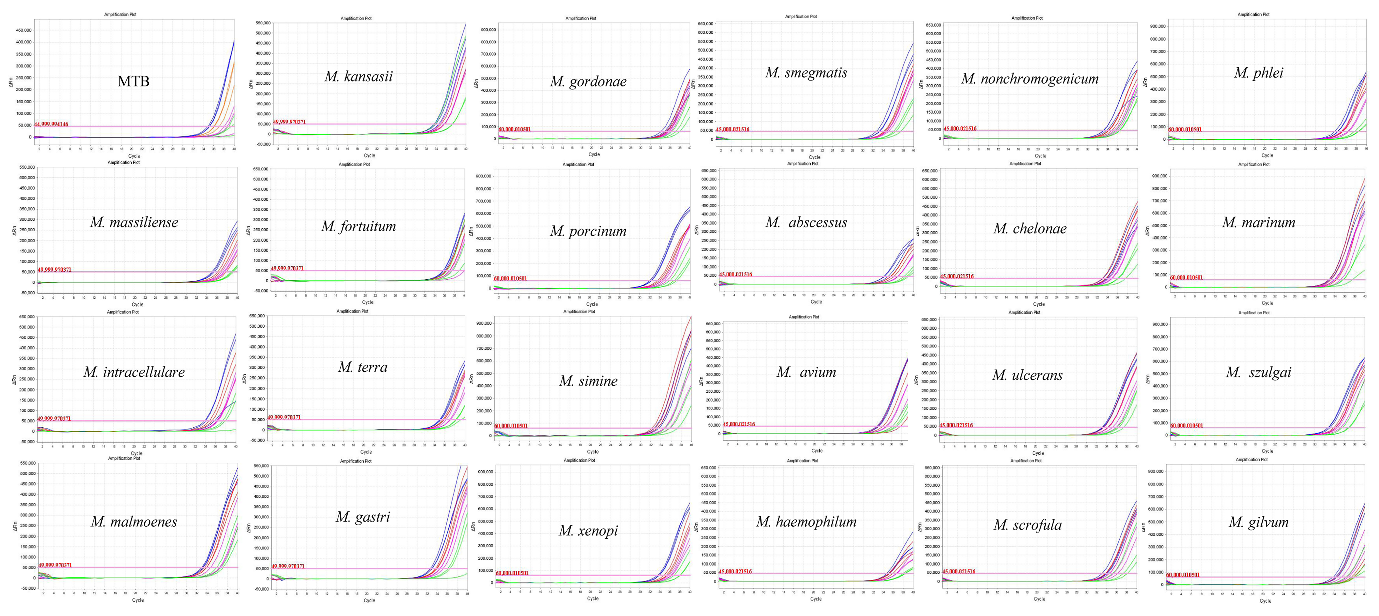


**Figure S2: Standard curves for defining the LOD in the Multiplex PCR MTB/NTM assay for *M. tuberculosis* and 23 NTM species.** Each panel of *M. tuberculosis* and 23 NTM species represents the amplification dynamics for a specific mycobacterium, denoted clearly by its respective label. The curves within each panel are color-coded to indicate four different concentrations of bacterial DNA: green (250 copies/ml), red (500 copies/ml), orange (1,000 copies/ml), and blue (2,000 copies/ml). These concentrations span from low to moderate, providing a detailed view of the assay’s sensitivity across a range of bacterial loads. Each condition was replicated in triplicates to ensure reliability of the data. The amplification cycle (x-axis) against fluorescence intensity (y-axis) plots are designed to provide a clear visual representation of the multiplex PCR amplification efficiency and the quantitative capabilities of the assay at various DNA concentrations. The LOD for each species was determined based on the lowest concentration at which all three replicates were successfully amplified.


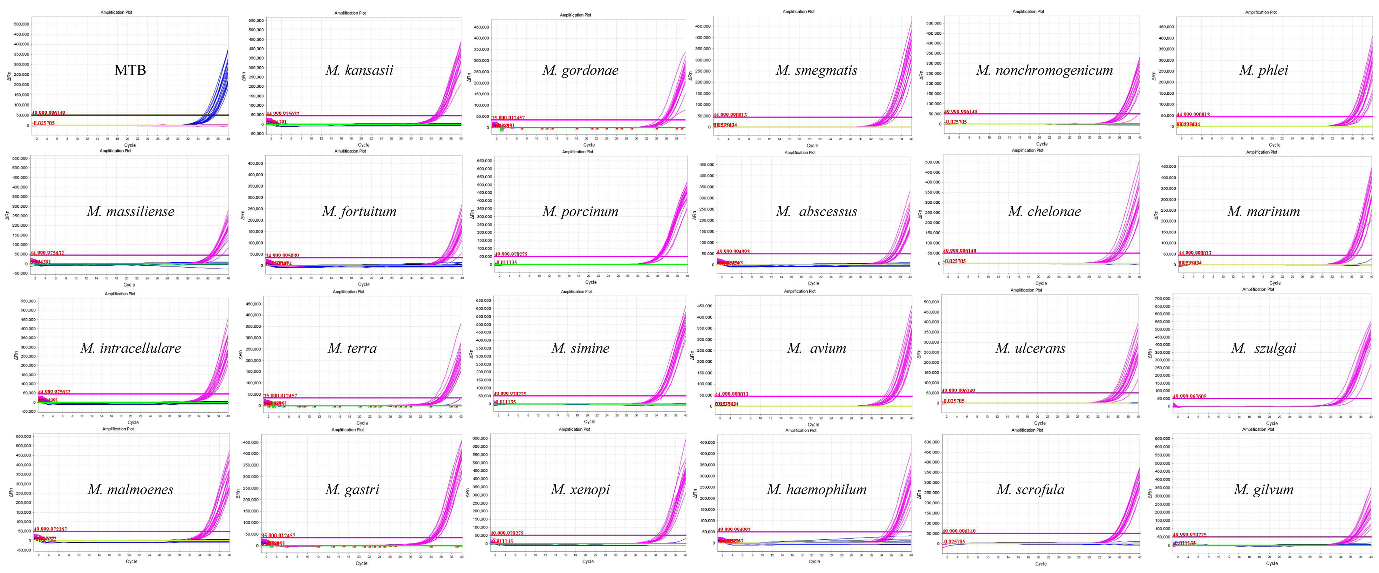


**Figure S3: Confirmation of LOD of Multiplex PCR MTB/NTM assay for *Mycobacterium* Species.** Each panel displays the amplification curves for 20 replicate tests of nucleic acid samples at a concentration of 10^3^ copies/ml measured by the Multiplex PCR assay. For *M. tuberculosis*, shown in the first panel, the consistent amplification across replicates indicates a robust detection capability of the assay at this concentration, revealed through the FAM fluorescence channel. Similarly, the subsequent panels represent the assay’s performance for each of the 23 NTM species as displayed through the Texas-Red Fluorescence channel. The tight clustering of amplification curves within each panel demonstrates the high precision and reliability of the assay in detecting *M. tuberculosis* and NTM species at the established LOD. The fluorescence intensity, depicted in the amplification curves, effectively demonstrates the assay’s sensitivity and consistency across all tested species. Each graph includes cycle numbers on the x-axis against fluorescence intensity on the y-axis, highlighting the exponential phase of DNA amplification critical for accurate diagnostics.

**Additional file 3: Validation of the Limit of Detection (LOD) of the *IS6110* primers-probe set in the Multiplex PCR MTB/NTM assay.**

**Methods**

The limit of detection (LOD) of our Multiplex PCR MTB/NTM assay for *M. tuberculosis* identification was previously established at 1000 copies/ml using DNA extracted from the reference *M. tuberculosis* strain H37Ra. To further validate this LOD, we utilized the Chinese national reference standard J10 for PCR-based *M. tuberculosis* (reference number: CMCC 93009, purchased from the National Institute for Food and Drug Control of China). For comparative analysis, H37Ra DNA was precisely quantified at 1000 copies/ml using droplet digital PCR (ddPCR), while the pre-quantified J10 national reference (1000 bacilli/ml) was diluted 10-fold to achieve a final concentration of 100 bacilli/ml, following the official guidelines for PCR assay LOD quantification. Both samples underwent identical DNA extraction protocols before amplification. The *IS6110* primers-probe set from our Multiplex PCR MTB/NTM assay was used to analyze 20 replicates of the H37Ra DNA (following our internal validation protocol) and 10 replicates of the J10 national reference DNA (as prescribed in the reference standard manual).

**Results**

The amplification plots for both the H37Ra strain (1000 copies/ml) and the J10 national reference standard (100 bacilli/ml) displayed comparable amplification kinetics, with threshold crossing occurring between cycles 35-37 (Figure S4, A and B). The Ct values for the H37Ra strain (n=20) had a mean of 36.26 with a standard deviation (SD) of 0.60 and a coefficient of variation (CV) of 1.66%. The National Reference Standard strain (n=10) yielded a mean Ct value of 35.90 with an SD of 0.46 and a CV of 1.27% (Table S6). Statistical comparison demonstrated no significant difference between these datasets (p>0.05), confirming equivalent quantification cycle values despite the samples' different origins and concentration units.

**Discussion**

The highly consistent amplification curves obtained from both the J10 national reference standard and H37Ra DNA validate that the LOD of 1000 copies/ml established using H37Ra accurately represents the assay’s performance with a certified reference material. This concordance demonstrates that the assay’s analytical sensitivity is comparable to the expected performance based on the reference standard’s defined concentration of approximately 100 bacilli/ml, thus confirming the robustness of our established LOD across different *M. tuberculosis* strains and reference materials.

Figure S4


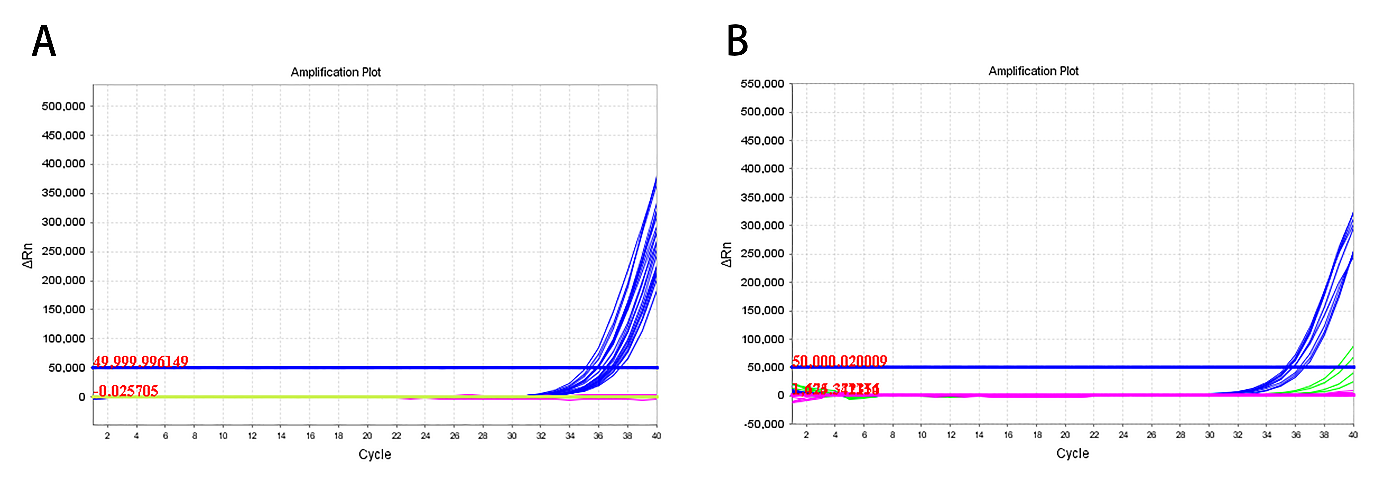


**Figure S4**: **Comparative Amplification Curves of *IS6110* PCR Assay Using H37Ra and National Reference J10 Strains. A)** Amplification curves for H37Ra strain (1000 copies/mL, n=20); **B)** Amplification curves for National Reference strain J10 (100 bacilli/mL, n=10).

Table S6: Analytical Performance Comparison of IS6110 PCR Assay Between H37Ra and National Reference J10 Strains at Their Respective Limit of Detection.

| *IS6110* PCR Assay against H37Ra strain for Validation of LOD | | | | | *IS6110* PCR Assay against National Reference strain J10 for Validation of Repeatability | | | | |
| --- | --- | --- | --- | --- | --- | --- | --- | --- | --- |
| Concentration | Ct of 20 replicates | Mean | SD | CV% | Concentration | Ct of 10 replicates | Mean | SD | CV% |
| 1000 copies/ml | 35.62 | 36.26 | 0.60 | 1.66 | 100 bacilli/ml | 36.51 | 35.90 | 0.46 | 1.27 |
|  | 37.13 |  |  |  |  |  |  |  |  |
|  | 35.36 |  |  |  |  | 35.32 |  |  |  |
|  | 37.04 |  |  |  |  |  |  |  |  |
|  | 35.92 |  |  |  |  | 35.85 |  |  |  |
|  | 35.85 |  |  |  |  |  |  |  |  |
|  | 36.42 |  |  |  |  | 35.47 |  |  |  |
|  | 35.86 |  |  |  |  |  |  |  |  |
|  | 35.83 |  |  |  |  | 36.28 |  |  |  |
|  | 36.74 |  |  |  |  |  |  |  |  |
|  | 36.80 |  |  |  |  | 35.61 |  |  |  |
|  | 35.2 |  |  |  |  |  |  |  |  |
|  | 35.81 |  |  |  |  | 35.88 |  |  |  |
|  | 36.82 |  |  |  |  |  |  |  |  |
|  | 37.10 |  |  |  |  | 36.67 |  |  |  |
|  | 35.86 |  |  |  |  |  |  |  |  |
|  | 36.59 |  |  |  |  | 35.51 |  |  |  |
|  | 36.94 |  |  |  |  |  |  |  |  |
|  | 36.21 |  |  |  |  | 35.87 |  |  |  |
|  | 36.16 |  |  |  |  |  |  |  |  |

Note: Statistical comparison revealed no significant difference in mean Ct values between the two groups (t = 1.86, df = 23.14, *p* = 0.08).
